# Unraveling the Amplification-Free Quantitative Detection of Viral RNA in Nasopharyngeal Swab Samples Using a Compact Electrochemical Rapid Test Device

**DOI:** 10.1021/acs.analchem.5c01605

**Published:** 2025-05-30

**Authors:** Manuel Gutiérrez-Capitán, Eva Balada, Anna Aviñó, Lluïsa Vilaplana, Roger Galve, Alícia Lacoma, Antonio Baldi, Antonio Alcamí, Véronique Noé, Carlos J. Ciudad, Ramón Eritja, María-Pilar Marco, César Fernández-Sánchez

**Affiliations:** † Instituto de Microelectrónica de Barcelona (IMB-CNM) CSIC, 08193 Bellaterra, Spain; ‡ Institute for Advanced Chemistry of Catalonia (IQAC) CSIC, 08034 Barcelona, Spain; § Centro de Investigación Biomédica en Red de Bioingeniería, Biomateriales y Nanomedicina (CIBER-BBN), Instituto de Salud Carlos III, 28029 Madrid, Spain; ∥ Institut d’Investigació Germans Trias i Pujol (IGTP), Camí de les Escoles, 08916 Badalona, Spain; ⊥ Centro de Investigación Biomédica en Red de Enfermedades Respiratorias (CIBERES), Instituto de Salud Carlos III, 28029 Madrid, Spain; # Centro de Biología Molecular Severo Ochoa, CSIC, and Universidad Autónoma de Madrid, 28049 Madrid, Spain; ∇ Department of Biochemistry and Physiology, School of Pharmacy and Food Sciences, University of Barcelona (UB), 08028 Barcelona, Spain; ○ Instituto de Nanociencia y Nanotecnología (IN2UB), University of Barcelona (UB), 08028 Barcelona, Spain

## Abstract

Providing viral load numbers of infection events aids in the identification of disease severity and in the effective overall patient management. Gold-standard polymerase chain reaction (PCR) techniques make this possible but cannot be applied at the point of need and in low-resource settings. Here, we report on the development of a compact analytical platform that can detect a conserved sequence of the RNA of severe acute respiratory syndrome-coronavirus 2 (SARS-CoV-2) in 40 min in nasopharyngeal swab samples without the need for any previous purification or gene amplification steps. It combines electrochemical and paper fluidic approaches together with a sandwich hybridization assay performed on magnetic nanoparticles (MNPs) modified with a tailor-designed capture DNA hairpin. The device proves to quantitatively detect viral RNA in a retrospective study carried out with nasopharyngeal swab samples. A sensitivity of 100% and a specificity of 93% were estimated by the receiver operating characteristic (ROC) analysis. However, although molar concentration values of the target RNA sequence are provided, these estimates do not fully correlate with the viral load numbers estimated by RT-qPCR over the whole Ct sample range. Empirical studies have been carried out that have provided clear insights into this hurdle and simple solutions to overcome it, without depriving the device of the features required for potential use in a point-of-care (PoC) environment.

## Graphical abstract

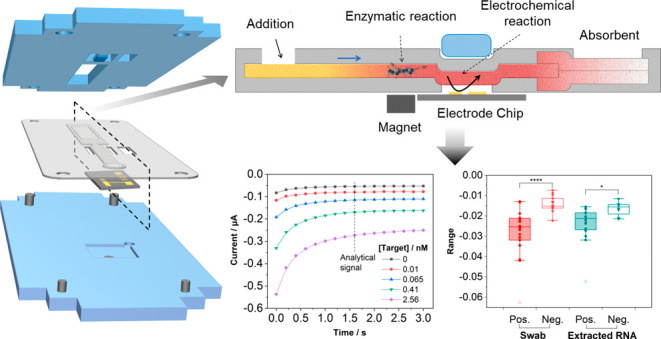

## Introduction

For more than 5 years, since the first reports of severe acute respiratory syndrome-coronavirus 2 (SARS-CoV-2) infection, the world has witnessed its devastating effects. Immense efforts were put to apply life-saving solutions that aided in reducing the pandemic economic and social impact.^1^ Although the World Healthcare Organization (WHO) announced the end of the emergency phase of COVID-19 in May 2023,^2^ there have been 777,385,370 confirmed cases, including 7,088,757 deaths, reported worldwide by 07 February 2025, with 66,305 cases and 3620 deaths reported in the last 28 days.^3^ Currently, only 67% of the world population has been vaccinated with a complete primary series, with the immunization percentage being especially low in the countries of the African continent.^4^ In addition, the virus continues to evolve to new Omicron sublineages, such as EG.5, unofficially nicknamed “Eris”, designated as a variant of interest by WHO on 9 August 2023,^5^ or JN.1, which is currently the most prevalent SARS-CoV-2 variant globally.^6^ That is why we still cannot consider the pandemic to be entirely over, and a shift to sustainable comprehensive management of COVID-19 within broader disease prevention and control programs has taken place. In fact, in its updated April 2023–April 2025 Strategic Preparedness, Readiness and Response Plan, WHO specifically details as a main objective the COVID-19 diagnosis through access and optimal use of safe and effective early diagnosis tools.^7^

The pandemic has clearly shown the shortcomings of our healthcare systems in the massive deployment of low-cost point-of-care (PoC) testing devices for pathogen detection. There was an urgent need for rapidly detecting virus infection, not only by identifying the infection agent but also by providing viral load numbers in order to give a more accurate information about the possible infection severity.^8^ For this, reliable, sensitive, and specific analytical tools that do not require any sophisticated equipment, complex measurement protocols, and tedious fabrication procedures should be developed. In this regard, decentralized nucleic acid molecular tests are at the forefront for closing this gap.^9,10^

The gold-standard analytical technology for COVID-19 diagnosis has been the reverse transcription-polymerase chain reaction (RT-PCR) for viral RNA detection.^11^ While this consolidated technique is able to provide sensitive and specific quantitative detection of SARS-CoV-2, it suffers from important limitations mostly related with the long analysis times (4–5 h) required for the initial nucleic acid extraction, amplification thermal cycling, and the costly instruments and reagents, as well as the need for skilled personnel.^12,13^ In this context, amplification-free approaches have introduced a new paradigm in molecular diagnostics where the viral RNA is directly detected, meeting the need for simple, rapid, and portable point-of-care (PoC) analysis.^14^ Although there are examples based on optical,^15^ colorimetric,^16^ electrochemiluminescent,^17^ and electrochemical^18^ signal generation, each of them showing pros and cons, the focus should be on achieving the best possible balance between high-performance analysis and producing a portable and miniaturized device for point-of-need applications.^19^ In this regard, electrochemical transduction approaches are particularly suitable because they offer superior sensitivity and accuracy, combined with the small size, low cost, low power consumption, and portability of the required electronics. Moreover, they are highly versatile as they can be manufactured in a wide range of different architectures to suit specific analytical detection schemes.^20^

Amplification-free electrochemical devices reported to date are based on well-known transduction modes applied to this particular application. In spite of the superior performance of most of the reported devices, these have only been evaluated with standard buffer solutions,^21,22^ spiked synthetic samples,^23–26^ or cell culture samples.^27,28^ However, measurements with clinical samples from COVID-19 positive and negative patients are necessary to produce sensitivity and specificity numbers that demonstrate the potential performance of the device in a clinical setting and enable comparative studies with other developed devices in development or in use for the same application. For these studies, nasopharyngeal swabs^29–33^ have been selected although other clinical specimens, such as sputum, urine, or plasma, have also been analyzed.^34^ It has been evidenced that the quantification of the viral load would aid in rapidly stratifying patients arriving at care units in order to timely apply an effective treatment.^35^

In this work, we show a simple paper-based electrochemical device to reliably detect a specific sequence of the SARS-CoV-2 viral RNA in untreated nasopharyngeal swab samples without requiring gene amplification steps and providing a response in 40 min. Paper has widely been the material of choice to produce fluidic approaches in PoC devices. It is a flexible, biocompatible, light-weighted, and porous material that enables liquid solutions to flow via capillary action without the need for any external pumping component.^36,37^ Our device combines a miniaturized two-electrode cell and a paper fluidic component designed to fit in a cartridge that allowed both parts to be easily aligned and to be replaced whether necessary. The detection is based on the implementation of a sandwich-like hybridization assay format performed on magnetic nanoparticles (MNPs) modified with a tailor-made polypurine reverse-Hoogsteen (PPRH) DNA probe that selectively captures the target RNA sequence. This in turn reacts with a reporter probe consisting of a DNA oligonucleotide conjugated to a horseradish peroxidase (HRP) enzyme, enabling the electrochemical readout of the target RNA sequence concentration in the sample. Although paper-microfluidic technology, electrochemical transduction, and enzyme-based affinity assays onto MNPs have been previously explored, this particular combination to specifically target oligonucleotides for amplification-free rapid detection of coronavirus RNA sequences has not been fully addressed,^38,39^ although it shows great promise as an analytical tool to be implemented at the point of need. A thorough analytical characterization of the resulting device is provided. Further, an in-depth study of the direct detection of the virus sequence in the target samples is included. This allowed us to critically assess the impact of the structure of the whole viral RNA on the sandwich hybridization process implemented for quantitative purposes, as well as to provide feasible solutions to overcome them.

## Results and Discussion

### Fabrication and Operation Principle of the Device

The device architecture presented in this study is shown in Figure 1. It comprises a compact paper-microfluidic electrochemical device that quantitatively detects a target sequence of the SARS-CoV-2 RNA with the required selectivity and sensitivity by exploiting highly specific PPRH-modified MNPs as the sensing element.

**Figure 1:**
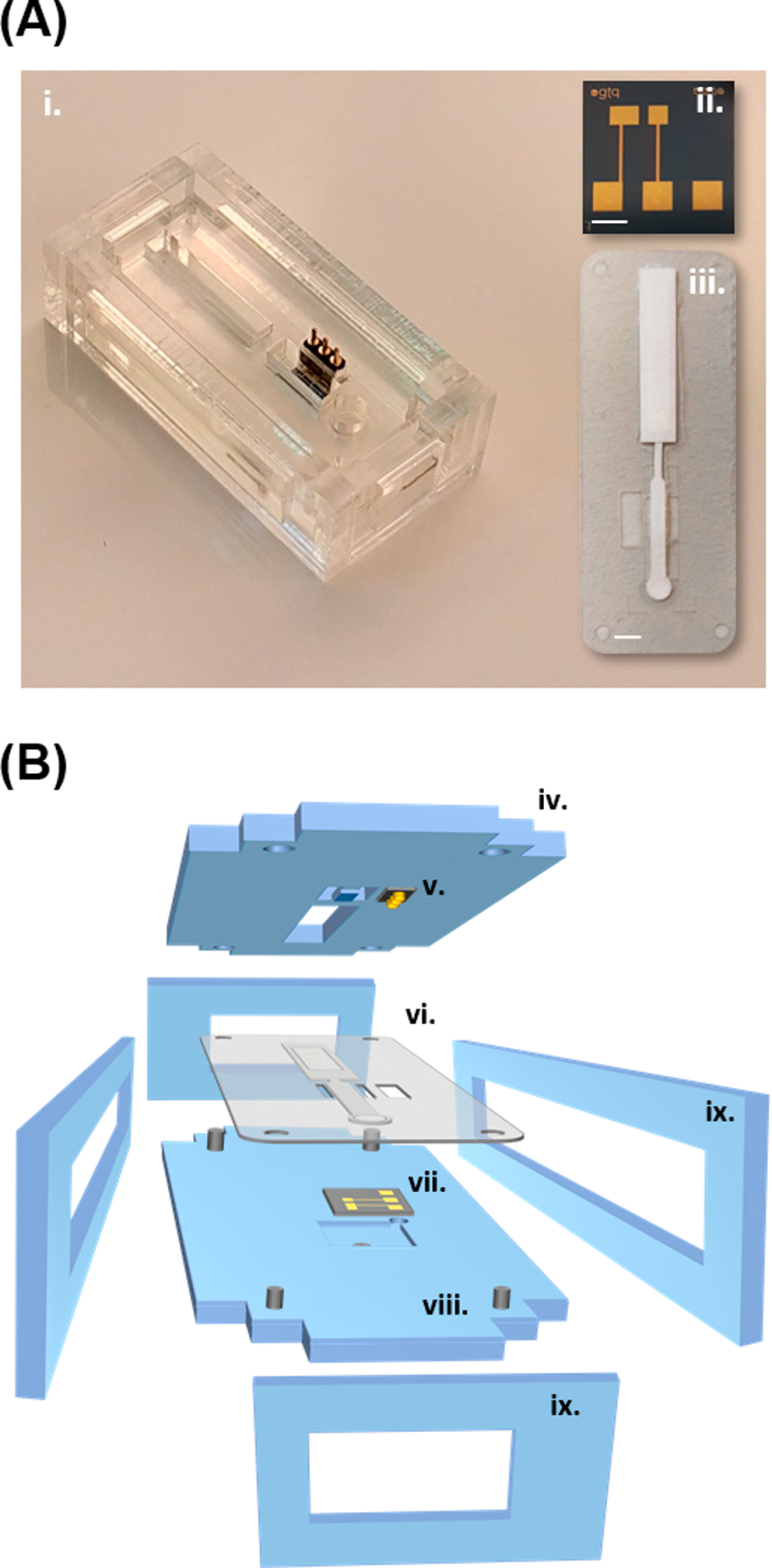
(A) Pictures of the device showing (i) the poly­(methyl methacrylate) (PMMA) cartridge where the top part, the clamping structures, and the spring-loaded connector are clearly visible; (ii) the two-electrode chip and, (iii) the paper component. Scale bars—chip: 2 mm, paper: 4 mm. (B) Exploded view of the device layout showing (iv) the top part, (v) spring-loaded connector, (vi) paper component, (vii) chip, (viii) bottom part, and (ix) clamping structures.

The electrochemical cell is based on a two-electrode configuration comprising a 1.5 mm^2^ counter/reference electrode (CRE) and a 1 mm^2^ working electrode (WE), both made of gold and patterned on an 8 × 8.3 mm^2^ silicon chip (Figure 1A). We have previously shown the excellent performance of this cell arrangement in the detection of activities of different enzymes whose reactions were coupled to reversible redox mediators^40^ and, more recently, by using horseradish peroxidase (HRP) as a label in magneto-immunoassays for the detection of chronic obstructive pulmonary disease (COPD) biomarkers in sputum.^41^ Likewise, other authors have further shown the benefits of applying similar compact electrochemical cell configurations for biomarker detection.^42–44^

The paper component is also shown in Figures 1A and S1 (Supporting Information (SI)). It comprises a single fluidic structure with four distinctive areas, all of them made of paper of chromatographic quality, commonly used in paper microfluidics.^45^ One paper piece includes a 4 mm circled solution addition pad, defined on one end of a 2 mm wide straight channel, which in turn shows a 1 mm narrower area at the opposite end of the fluidic channel, designed with the aim of better controlling the flow rate in the fluidic structure.^44^ A second paper piece defines a 5 × 50 mm^2^ sink rectangular pad, which is bent at the middle and overlaps the fluidic channel on both paper sides. Different paper patterning approaches have previously been reported.^46^ Among them, die cutting is very convenient for mass producing simple geometric features. The application of this technique entailed the design and fabrication of a die including 17 repeated units of the two paper pieces and the eventual paper cutting using a custom-made manual die cutter. Once fabricated, the die could be repeatedly used without wear.

Each die cutting process lasts around 10 s, yielding very well-defined paper pieces with excellent reproducibility. The correct arrangement of both paper pieces and the placement over the on-chip electrochemical cell was facilitated by sandwiching them between two sticky vinyl layers. These were designed to include several opened windows to access the paper in the sample addition, electrode, and evaporation areas as well as to allow contacting the on-chip contact pads by the spring-loaded connectors (more details in the SI). These vinyl layers were also cut with a custom-made die, obtaining 9 vinyl components for each cutting process, which lasts around 10 s, too.

The polymeric cartridge is arranged so that the transducer and paper fluidic components can easily be inserted and aligned (Figure 1A,B). The bottom layer includes a recessed area to host the chip and an embedded 2 mm diameter thick Nd magnet to trap the magnetic nanoparticles in a specific area of the paper component. The top layer includes two open areas for allowing the addition of solutions on the paper pad and for favoring the solution evaporation of the sink pad. It also comprises a flexible bridge structure that protrudes from the PMMA used to press the paper channel over the electrodes, thus guaranteeing the intimate contact between these two main components in the detection area of the device. Four rigid clamping structures making a frame are included for tightly keeping both top and bottom parts in place. They also facilitate the rapid assembly and disassembly of the device, when necessary.

Direct biomarker detection in complex biological samples with microfluidic-based POCT has proven to be difficult. The geometrical arrangement of lateral flow devices, including areas for retention of sample components and integration of reagents,^36^ appears to facilitate such analyses but in most cases at the cost of device-limited sensitivities and high limits of detection (LD).

The detection of SARS-CoV-2 virus at different stages of infection required low limits of detection (LD) and virus RNA extraction, followed by target sequence amplification using PCR detection approaches was mainly used in this regard. An excellent alternative was to implement a molecular assay based on MNPs, which play a double role. In an initial step, MNPs were applied for RNA capture and separation from the sample outside the device (Figure 2A). For this, MNPs modified with a PPRH complementary sequence RNA were incubated in the sample together with an HRP-conjugated reporter sequence. The captured RNA was thus labeled with HRP. The modified MNPs were then rinsed and resuspended in a buffered solution before being added to the paper electrochemical device. At this stage, the MNPs enabled the accumulation of the HRP-labeled RNA on the device before carrying out the electrochemical detection. This was possible thanks to the Nd magnet in the cartridge, located below the paper channel, 1.5 mm upstream from the position of the working electrode. The diameter of the MNPs was selected after testing their flow on the chromatographic paper and their efficient capture by the Nd magnet. The complete analytical procedure comprised five steps: (i) simultaneous incubation of the sample/standard solution with the functionalized MNPs and the HRP-conjugated reporter sequence, (ii) MNP resuspension in a buffer solution, (iii) MNP addition to the device inlet, (iv) paper channel washing, and (v) addition of the HRP substrate solution to carry out the eventual chronoamperometric detection (Figure 2B). All of these steps simply required pipet manipulation for which no specific training is needed.

**Figure 2:**
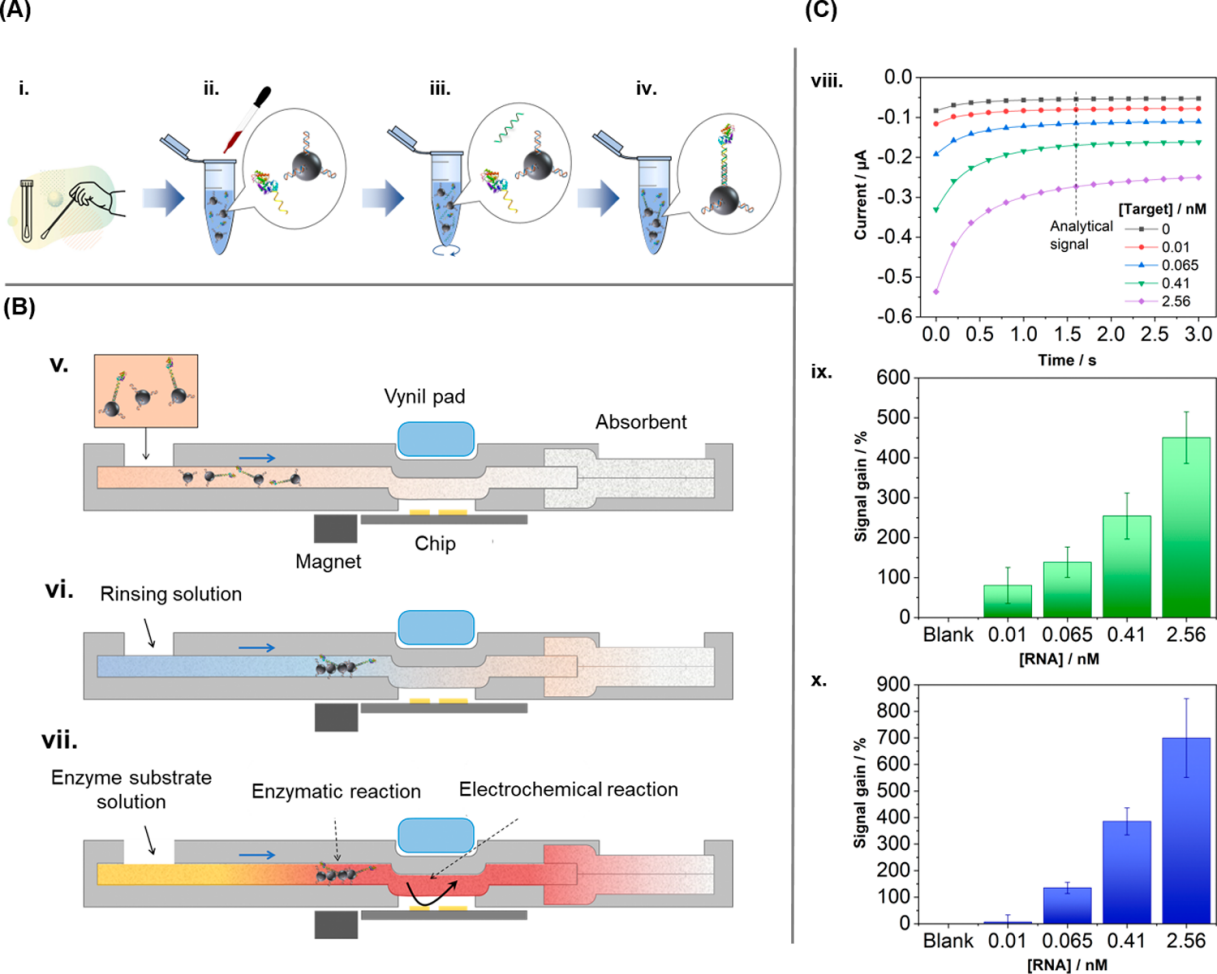
Analytical performance of the device. Schemes in panels (A, B) showing incubation of the sample with MNP-PPRH and HRP-SC to capture the RNA target analyte: (i) sample collection; (ii) sample addition; (iii) sample incubation; (iv) capture of target sequence, and sandwich hybridization assay (drawing of the hybridization reaction shown in Figure S2A, SI); (v) MNPs added to the fluidic device after capturing the RNA target analyte and allowed them to flow through the paper and be trapped by the magnet; (vi) rinsing step to flux all of the MNP toward the magnet; (vii) addition of the Fe-MeOH enzyme substrate, corresponding enzymatic reaction, and electrochemical detection (enzymatic and electrochemical reactions shown in Figure S2B, SI). Drawings not to scale. Analytical data in panel (C). (viii) Chronoamperometric responses to different concentrations of the RNA target sequence in standard buffer solutions; (ix) bar graph showing the increase of the device signal with the RNA target concentration in buffer solutions containing the same concentrations as in (viii); (x) the same as in ix but the RNA target is in universal transport media (UTM). Signal gain refers to ((ic – ic_0_)/ic_0_) × 100, ic being the current recorded for a given RNA concentration and ic_0_ being the current recorded in the blank solution, that is, the one that does not contain the RNA target sequence. Error bar represents the standard deviation of three measurements.

### Magnetoassay Studies

The detection of the SARS-CoV-2 RNA was based on a sandwich hybridization assay format using the MNPs functionalized with the PPRH capture DNA sequence to the complementary target RNA sequence (MNP-PPRH) and the use of a reporter DNA sequence labeled with the HRP enzyme (HRP-RS). Optical and electrochemical detection protocols were applied by measuring the activity of the enzyme label in solutions containing H_2_O_2_ substrate and an appropriate enzymatic redox mediator. From the different RNA sequences that were assessed to be potentially used in this device together with the corresponding PPRH capture sequences,^47^ one of them was selected, which is located at the 17,143 starting nucleotide position, this being a fragment of the gene that encodes the helicase nonstructural protein 13. This sequence targets the region encoding the 5620-5626 amino acids of the ORF1ab where no mutations have been identified in any of the reported variants of the virus (α, β, γ, Delta, Omicron including the Eris and JN.1 subvariants).^48^

The sandwich hybridization assay was optimized in univariate assays. A detailed description is included in the SI (Figure S3). Two-step and one-step assay formats, meaning the respective sequential or simultaneous incubation of the target sequence with the functionalized MNPs and with the HRP-conjugated reporter sequence, were tested. As can be seen in Figure S3C, SI, there were no statistical differences between the results achieved with both assay formats. Therefore, the one-step-based assay was performed. The overall assay time was shortened by 5 min by removing the two manual steps. For a concentration range of 0.01–16 nM, the regression coefficient (*R* ^2^, three replicates) obtained for the one-step curve fitting performed using a dose–response semilogarithmic approach was 0.990, and the estimated limit of detection (LOD) was 0.043 nM (3σ IUPAC criterion). These optimized conditions were then used in the implementation of the magnetoassay on the electrochemical device.

### Analytical Performance of the Electrochemical Device

Electrochemical measurements were based on the use of the ferrocenemethanol (Fc-MeOH) redox mediator of the HRP label (Figure S2, SI). The corresponding catalytic oxidation of the H_2_O_2_ enzyme substrate produced ferrocinium-methanol cation ([Fc-MeOH]^+^) that was detected by chronoamperometry at −0.15 V. At this potential value, [Fc-MeOH]^+^ was reduced back to Fc-MeOH, using the two-electrode electrochemical cell.^41^

The analytical performance of the paper-microfluidic electrochemical device was first assessed in standard hybridization buffer solutions. The raw chronoamperometric responses to the target sequence concentrations and the blank signal were recorded. As expected, the cathodic currents increased with the target oligonucleotide concentration, 0.01 nM (1 fmol) being the lowest concentration providing a signal that differed from the blank (Figure 2C). The corresponding calibration curve was constructed using the current values recorded at 1.6 s as the analytical signal (Figure 2C). A semilogarithmic dose–response fitting in the concentration range studied of 0.01–2.56 nM was carried out (*R* ^2^ = 0.950, three replicates) and used to estimate the LOD, which was 0.018 nM (3σ IUPAC criterion). The overall time of analysis was around 40 min. Higher RNA target concentrations were studied (16–100 nM), showing a leveled-off or even a decreased current signal. The latter might be related to the Hook effect that, to a certain degree, is known to take place at high concentration values when working with oligonucleotide hybridization reactions.

A study of the sample matrix effect on the analytical signal was carried out using universal transport media (UTM) in order to simulate the real conditions of analysis. Figure 2C shows the bar graph comparing the device response to increasing concentrations of the target sequence in this UTM. The estimated LOD using the same fitting as above (*R* ^2^ = 0.994, three replicates) was 0.076 nM. The graph and this value indicate that the UTM, which contains an inactivating agent, interfered to some extent with the hybridization reactions of the oligonucleotide sequences. Nevertheless, a similar response trend to that observed in the standard hybridization buffer solutions was observed for the concentration range of the tested target sequence.

The achieved limits of detection may appear to be not low enough for the amplification-free analysis of clinical samples. However, these calibration studies were performed with synthetic DNA target sequences, whose dissociation constants with the hairpin PPRH capture probes appear to be over 4 times higher than those observed with the corresponding RNA target sequence. This study has previously been reported by some of the authors of this work.^49^ Such an effect was further confirmed by the analysis of viral RNA from cell extracts, the results of which are presented in the Quantitative Analysis of SARS-CoV-2 RNA in Clinical Human Samples section. Moreover, the retrospective study of clinical samples described below fully proved it.

### Analysis of Clinical Samples

The retrospective analysis of 58 nasopharyngeal swab samples, 36 positive and 22 negative, confirmed by PCR, was carried out with the electrochemical device. The samples were split into two groups. The first group included 34 samples that were collected and directly frozen before the analysis, and the second group included 24 samples where the RNA was extracted and then frozen before the analysis (more details can be found in the SI. The UTM used for sample collection contained an inactivating agent to make sure that the sample was not infective.^50,51^ Samples were tested in sets, and the analyses with the electrochemical device entailed the additional measurement of blank and control samples prepared in the same UTM, the latter containing 0.41 nM of the synthetic DNA oligonucleotide, to assess the correct performance of the device. The two-sided *t* test carried out with both swab and extracted RNA samples shows significant differences in the electrochemical device responses for negative and positive samples, these being highly different for the swab samples (*P* = 0.00002) and more limited for the extracted RNA ones (*P* = 0.036) (Figure 3A). The receiver operating characteristic (ROC) curves constructed for the two groups of samples corroborated these findings. The areas under the curve (AUCs) for the swab and extracted RNA samples were 0.98 and 0.84, respectively (Figure 3B). A sensitivity of 100% and a specificity of 93% (95% confidence interval, −18.3 nA cutoff value) were achieved in the analysis of the swab samples with the presented device. Surprisingly, samples of extracted RNA produced a less appealing result. A sensitivity of 81% and a specificity of 75% were calculated for an optimum cutoff value of −17.7 nA. This effect may be related to the different kits used for the RNA extraction and the overall sample processing.^52^ It has previously been reported that several commercial kits produced different RNA extraction yields, and this was dependent on the reagents and overall procedure behind the extraction process. However, what seems more relevant is that the quality of the extracted RNA, in terms of molecular integrity, could also differ, meaning that a bias in the quantification of RNA could be produced.^53^ Moreover, sample manipulation and medium-term storage of RNA-extracted samples in the freezer may degrade the samples to a certain extent, with this having a clear influence on the analyses performed with our device.

**Figure 3:**
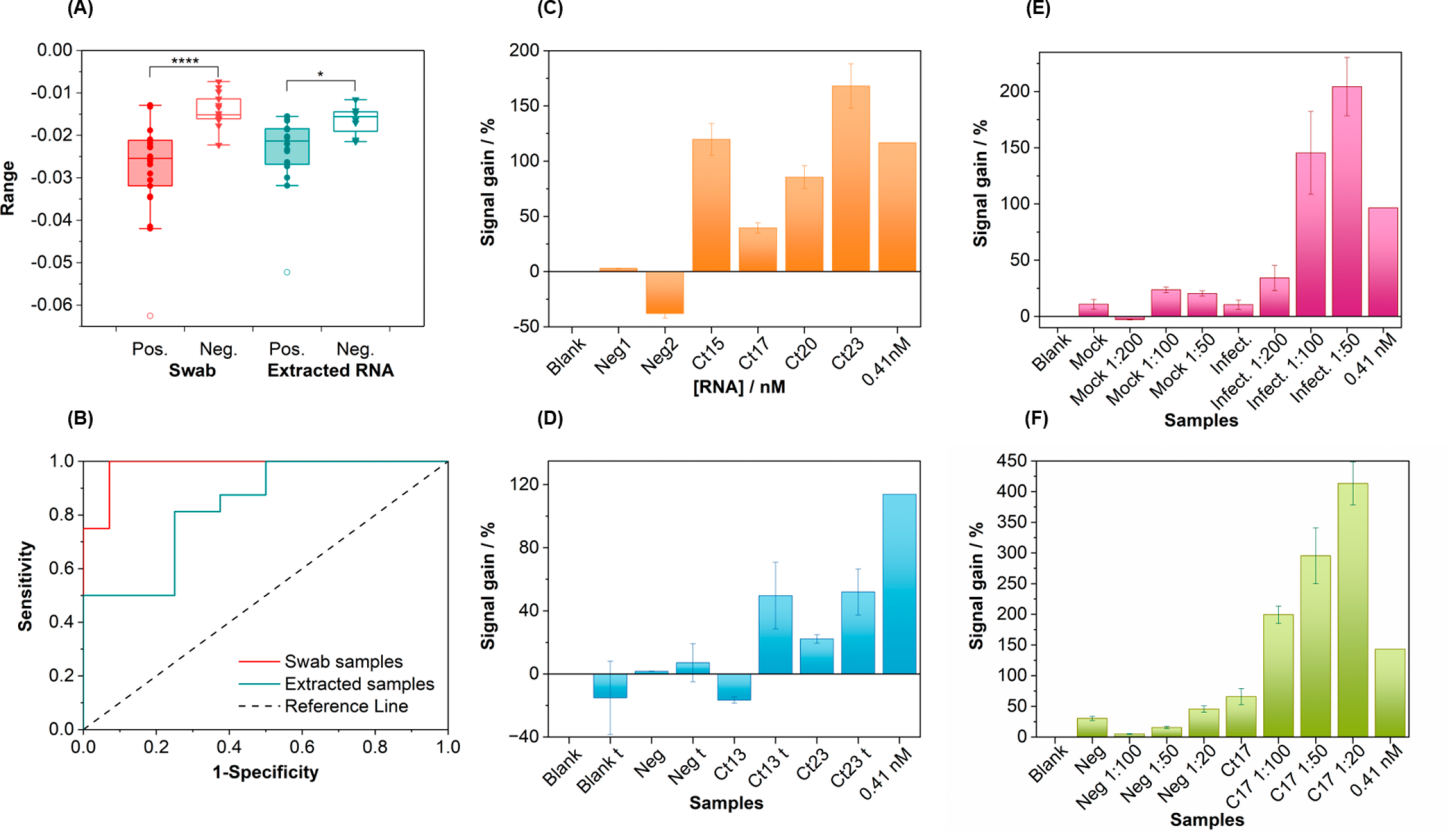
Retrospective analysis of two sets of clinical samples: 34 nasopharyngeal swab samples and 24 samples of extracted RNA. (A) Box and whisker graphs showing the difference between samples tested positive and negative by RT-PCR from the two sets. Boxes show Q1, Q3, and median values. Vertical lines describe the 1.5-quartile range (IQC). Empty symbols are samples categorized as outliers. *P* < 0.0001 swab samples; *P* < 0.5 extracted RNA. (B) ROC curves for the two sets of samples. Retrospective analysis of two representative small sets of samples, panel (C) being raw nasopharyngeal swab samples and (D) samples with disparate Ct values treated with the RNA fragmentation kit. Analysis of (E) mock and infected cell lysates and their serial dilutions (stock of infected cells contained 3 × 10^6^ RNA copies/μL) and (F) one positive and one negative sample and their serial dilutions. The letter “t” in the *X* axes of panels (D–F) refers to the treated samples. Error bars represent the standard deviation of three measurements. The signal corresponding to a 0.41 nM CC1 concentration was measured as a internal standard to assess the correct performance of the device.

The gold-standard approach for diagnosing COVID-19 has been the polymerase chain reaction (PCR) test that involves sample collection by a healthcare worker and sample transport to a clinical laboratory for performing RNA extraction/purification and further analyses. The overall time from the collection of the sample to the report of the results may exceed 24 h. Our approach shows the potential for taking the COVID-19 molecular diagnosis to the point of need, offering several practical advantages, such as the following: (1) the methodology is based on the combination of well-established technologies such as paper microfluidics, MNP-based assays, and very simple electrochemical detection; (2) liquid volumes including 100 μL of sample, reagent, and rinsing solutions are low, and this is due to the low number of manual steps that should be carried out; and (3) the assay time being around 40 min is significantly shorter than that of the PCR, mainly because RNA extraction, purification, and amplification steps were not required, thus making it appropriate for on-site diagnostics. In addition, the device relies on very simple, cost-effective, and low-power electrochemical techniques, and it could be used by any personnel after going through minimal training. All of these advantages translate into a low cost per analysis, which was estimated to be below 1 €.

Our approach meets some of the criteria set by the World Healthcare Organization (WHO) for priority diagnostics of COVID-19.^54^ WHO has defined the target product profile (TPP) that specifies the key features a diagnostic tool should fulfill in order to be applied in this context. A short version of the TPP is included in the SI (Table S1, SI). Among the different priority use-case scenarios considered by WHO, the device presented in this work may lay within the point-of-care tests for suspected COVID-19 cases and their close contacts to diagnose acute SARS-CoV-2 infection in areas where molecular/reference assay testing is unavailable or involve long turnaround times that are not useful for guiding clinical case management and infection control measures. The TPP includes acceptable and desirable features that a diagnostic platform should show for application in this scenario. Among them, it sets acceptable/desirable sensitivity and specificity values of ≥80/97 and ≥90/99%, or acceptable/desirable time to result values of ≤40/20 min, respectively. The TPPs published by WHO for COVID and other infectious diseases are aligned with the ASSURED concept also coined by WHO in 2003 through the special program for research and training in tropical diseases (WHO/TDR) that benchmarks the criteria a diagnostic test should address to be implemented in disease control programs. ASSURED accounts for Affordable, Sensitive, Specific, User-Friendly, Rapid, Equipment-Free or simple devices.^55^ Later in 2019, the REASSURED concept was published that included the digital component (real-time connectivity) and ease of specimen collection feature.^56^ Very recently, the REST-ASSURED concept appeared for case scenarios in resource-limited settings, where the ST letters account for scale-up manufacturing and distribution of diagnostic tests and transferability of assays to existing platforms (versatile technology), data and technology for a more equitable and in turn sustainable device production process.^57^

Our device ticks most of the acceptable key features of the TPP as well as the criteria defined in the ASSURE-derived concepts, thus holding promise to be applied in the molecular diagnosis of this disease.^58^ This analytical platform, when integrated with portable instrumentation, could potentially be used as a POCT device in low-resource settings. The ability of the designed sandwich oligonucleotide hybridization to detect the presence of viral RNA in a rapid and cost-effective way also makes the device of potential use in home and emergency units. The device could be controlled by a compact potentiostat, and some instrument approaches are already commercially available. These can be connected to a mobile device and battery-powered. The use of a custom-made app could provide direct results of the RNA viral load and help store the results and send them.

### Quantitative Analysis of SARS-CoV-2 RNA in Clinical Human Samples

There has been a clear need for accessing quantitative diagnostic platforms at the point of care that aided in the efficient patient stratification and timely effective treatment.^59^ Our main aim was to address the quantitative detection of the SARS-CoV-2 virus in nasopharyngeal swab samples by carrying out the retrospective analysis described in previous sections where the values provided by our diagnostic platform were compared with the viral load numbers obtained by RT-qPCR. Indeed, all of the analyzed samples were provided with the corresponding cycle threshold (Ct) values. This value defines the number of cycles of viral RNA amplification performed to reach a detectable RNA concentration. Therefore, Ct values are inversely proportional to the viral load in the sample and, hence, to the analytical signal of our electrochemical device. This correlation was observed with samples showing Ct values above 20. However, this was not observed for those samples with higher viral loads. Figure 3C shows the results of the analyses of a small batch of positive and negative samples with Ct values between 15 and 23. As can be observed, the signal gain values of samples tested negative by the PCR test were of the same order of magnitude to that of the blank. However, the positive sample with the highest Ct (23) among all the sample of the batch provided the highest analytical signal compared to the positive sample with a Ct 15. The same occurs with the samples with Ct values of 20 and 17. Similar results were often observed in other sets of samples. We hypothesized about the combined effect of the hybridization assay format and the RNA arrangement in the sample, mainly related to the position of the target sequence and the molecular structure of the RNA together with the intermolecular interactions that may take place. To shed some light on it, some of the analyzed samples were treated with a RNA fragmentation kit based on the use of zinc acetate reagents, which randomly cleaves phosphodiester bonds of the nucleotide backbone. This fragmentation process is often recommended with RNA samples that have been amplified before a hybridization assay on oligonucleotide microarrays. RNA fragmentation appears to improve the hybridization kinetics and thus the output signal by making the target sequences more accessible to the PPRH capture oligonucleotide hairpin immobilized on the MNPs.^60^ As can be seen in Figure 3D, the two positive samples treated with the kit showed an enhanced signal , while the change in the signal values of the negative sample and the blank was negligible. As expected, the signal increase in the sample with the highest viral load (Ct = 13) was greater than 60%, while for the sample with Ct = 23, it was only 25%. Working with such a protocol may improve the correlation between the PCR results and those recorded with our device. However, it should be considered that the concentration of the Zn reagent and the reaction time must be set depending on the RNA concentration in the sample in order to avoid the excessive degradation of the viral RNA that would produce the opposite effect on the analytical signal. Careful selection of the experimental conditions for controlled cleavage of RNA could be set in advance for specific viral load ranges of the samples in order to circumvent such a drawback.

From these results, it can be ascertained that making the target sequence more accessible to the functionalized MNPs improved the performance of the affinity assays and, thus, the detection carried out with the electrochemical device. Considering the secondary and 3D structures of the SARS-CoV-2 RNA (Figure S4, SI), the nsp13 coding region where the target sequence is located shows a complex structure including bulge, hairpin, internal, and multibranched loops, together with stems.^61–63^ These structure may be steric hindrances that might difficult the access of the capture hairpin oligonucleotide located on the surface of 200 nm diameter MNPs to reach the target sequence . This may partly explain the lack of correlation between the Ct value and the quantitative electrochemical signal recorded with the presented device. It should be mentioned at this point that the polypyrimidine target sequence was chosen because it can form high-affinity triplexes with the PPRH capture oligonucleotide,^47,64,65^ which clearly improved the sensitivity of the assay. In a previous work by some of the authors of this work, it was shown that the dissociation constant of using the triplex probe was two times lower than that estimated using the one forming the usual duplex structure with the target sequence. Such an apparent difference enhanced the limit of detection of the assay by 2 orders of magnitude.^66^

Looking at other approaches that could be more easily implemented in the device than the one described above, and with the aim to find a better correlation between the Ct values and the response of the electrochemical device in the whole Ct range of the analyzed samples, the calibration of the device performed with solutions containing a set amount of viral RNA was considered. However, no commercial standard solutions of SARS-CoV-2 RNA could be found having a concentration high enough to be used for this purpose. Nevertheless, genomic RNA coming from the culture supernatant of SARS-CoV-2/2021/FR/7b with a 1.25 × 104 copies/μL concentration (European Virus Archive—Global, EVAg) and a SARS-CoV-2 RNA control from a Spanish clinical isolate, with 1.3 × 104 copies/μL concentration (Vircell, SL, Granada, Spain), were tested, and neither of them provided an analytical response. Alternatively, a concentrated RNA stock solution derived from infected cell cultures was used. Once the cells were lysed and the viral RNA was released, the sample contained 3 × 10^6^ copies/μL (Ct = 9.87). Also, a mock cell solution, this being the supernatant of a cell culture of uninfected cells that were treated under the same conditions as the infected ones, was used in order to assess the matrix effects. As can be seen in Figure 3E, the estimated signal gain obtained with the mock cell solution, measured in triplicate, as well as the ones measured with the 1:50, 1:100, and 1:200 dilutions made with the hybridization buffer, are practically the same. In addition, the valuest did not differ from that obtained in the blank solution, clearly showing that there were no matrix effects. When measuring the sample that contains 3 × 10^6^ copies/μL viral RNA, a similar signal gain to that of the mock cell solution was obtained. By contrast, signal gain values obtained with the sample previously diluted 1:50, 1:100, and 1:200 showed a clear correlation with the viral load; that is, the response of the electrochemical device was inversely proportional to the sample dilution and then directly proportional to the SARS-CoV-2 RNA concentration. Thus, the 50-fold sample dilution produced the highest analytical signal, whereas the 200-fold dilution provided a signal similar to that of the mock cell solution. The 50-fold dilution translates into an RNA concentration of 50 fM, equivalent to 5 attmol in the 100 μL sample volume that was analyzed. These tests further corroborate the sensitivity of the presented device and the difference observed when the target sequence was DNA, used for the initial tests, or RNA. As mentioned above, we believe this effect was related to the dissociation constant of the PPRH capture oligonucleotide hairpin being 4 times lower for RNA than that estimated for its corresponding DNA sequence.^49^

The same study was carried out with two representative nasopharyngeal swab samples previously analyzed. As can be seen in Figure 3F, the sample tested negative by PCR and its serial dilutions produced similar signals, these being of the same range as that of the blank solution. In addition, the sample tested positive by PCR, with Ct 17 producing a rather low analytical signal, not significantly different from those of the blank and negative sample, as it had already been observed in the experiments with real samples, previously described. However, signals recorded with the analyzed sample dilutions show a direct correlation with the viral load, in the same fashion as in the study performed with the infected cell culture lysates.

From these results, we believe that interactions between RNA molecules that may take place to a higher degree at high concentrations produce a greater steric hindrance to the interaction between the target RNA sequence and the capture oligonucleotide immobilized on the MNPs, giving rise to a much lower analytical signal than expected. Sample dilution may alleviate this effect, making the target sequence more accessible for the functionalized MNPs, as happened when fragmenting the RNA with the Zn reagent. Likewise, it may eliminate the Hook effect that was apparent during the device calibration studies at high concentrations of the RNA target sequence.

All of these studies evidence a clear drawback that should be circumvented in the potential implementation of the device as a POCT. A feasible solution may be to carry out a dual measurement, that is, one with the real sample as it is and the other with a dilution of the sample. If the signal of the diluted sample is higher than that of the undiluted sample, it would be a clear indication that the viral load is high. By contrast, if the recorded electrochemical signal decreases, it would mean that a sample with a low viral load is being analyzed. Depending on these initial results, those of higher viral load could be analyzed again considering an initial sample dilution to more accurately assess the concentration of the target RNA. This may increase the overall analysis time but would clearly provide reliable quantitative information for a rapid diagnosis and prognosis in the evolution of the disease.

Comparative performance was obtained with previously reported devices. Table S2, SI, summarizes some of the functional features of the amplification-free electrochemical approaches validated with real clinical samples that, to the best of our knowledge, have been reported so far. The advantages of the paper-based electrochemical platform presented in this work include its feasible cost-effective production, ease of operation, and lack of complex equipment. The ease of operation should be highlighted since no RNA extraction/purification processes in the collected samples were required. By contrast, all of the device approaches that have been checked required a treatment of at least 30 min in order to inactivate the virus and purify the viral RNA. In addition, a total of 34 nasopharyngeal samples were analyzed with our device, a representative number if it is compared with the reported approaches working with this type of samples. A short analysis time of less than 40 min is one of the main features of the current device. Sensitivity and specificity values of 100 and 93%, respectively, achieved with intact collected samples are also highly relevant. Moreover, the presented device is the only one that addresses the potential correlation between the quantitative detection of RNA and the viral load Ct values provided by the PCR.

## Experimental Section

### Reagents and Solutions

All reagents used were of high purity, analytical grade, or equivalent and were purchased from Sigma-Aldrich (Madrid, Spain) unless stated otherwise. A detailed list is provided in the SI.

The target RNA sequence, expressed as the homologous synthetic DNA sequence used for performing the assay, was 5′-GAGTCATTTTGCTATTGGCCTAGCTCTCTACTACCCTTCTGCTC-3′. It starts at the 17143-nucleotide position of the RNA virus genome and is a fragment of the gene located at the ORF1b region that encodes the helicase nonstructural protein 13 (nsp13).^62^ Two oligonucleotides were used to perform the affinity assay. These were a polypurine reverse-Hoogsteen (PPRH) hairpin^64,65^ (5′-NH2-TTTTTGAGCAGAAGGGTAGTAGAGAGTTTTGAGAGATGATGGGAAGACGAG-3′) capture sequence that forms a triplex with the RNA of the virus and the thiol-modified 5′-GGCCAATAGCAAAATGACTC-Thiol-3′ reporter sequence. All of them were obtained either from commercial sources (Merck/Sigma-Aldrich, Haverhill, UK) or synthesized on an automatic H-8 DNA/RNA synthesizer (K&A Laboratories, Germany) on a 1 μmol scale using commercially available chemicals. The experimental details of the synthesis, as well as the complete characterization and evaluation of these oligonucleotide sequences, have previously been reported.^47^

### Electrochemical Cell and Paper Fluidic Component

A reusable electrochemical cell of two gold thin-film electrodes was fabricated by a standard photolithographic/lift-off process on 4-in. silicon wafers at the IMB-CNM Clean Room facilities.^67^ 8 × 8.3 mm^2^ silicon chips, each one including a 1 × 1 mm^2^ working electrode (WE) and a 1.5 × 1 mm^2^ counter/reference electrode (CRE), were manufactured (Figure 1B). More information is provided in the SI.

The different parts of the paper component were designed using CorelDRAW software (Corel Inc., Austin, TX) and cut using a custom-made die cutter (Tecnocut, Barcelona, Spain). A detailed description of the production process is provided in the SI.

### Cartridge Assembly

A poly­(methyl methacrylate) (PMMA) cartridge to integrate and align the electrochemical cell and the paper component was also designed with CorelDRAW software. PMMA substrates were machined using a CO_2_-laser printer (Epilog Mini 24, Epilog Laser, Golden, CO) (Figure 1B). A detailed description of this structure is provided in the SI.

### Functionalization of MNPs and Thiol-Reporter Sequence Conjugation

200 nm diameter MNPs were modified with the PPRH capture probe by covalent linkage between the MNP carboxylic groups and the 5′-terminal primary amine moieties of the oligonucleotide structure using the well-known carbodiimide chemistry.^68^ The detailed functionalization is provided in the SI.

The thiol-modified reporter sequence was labeled with the horseradish peroxidase (HRP) enzyme using an HRP-oligo conjugation kit (thiol oligo) from CellMosaic, Inc. (Woburn, MA).^69^ The details of the labeling steps are provided in the SI.

### Optimization of the Magnetoassay

The optimum conditions of the magnetoassay were set by performing univariate assays. A detailed description of these studies is provided in the SI.

### Analytical Performance of the Electrochemical Device

An EmStat potentiostat (Palmsens BV, Houten, The Netherlands) controlled by PSTrace v5.5 software was used for all of the chronoamperometric measurements. These measurements were based on the detection of the electrodic reactions undergone by ferrocenemethanol/ferrocinium-methanol (Fc-MeOH/[Fc-MeOH]^+^) redox pair. Fc-MeOH was used as the redox mediator to measure the activity of the HRP label. A detailed description of the analytical steps performed for every single measurement is provided in the SI.

The HRP label catalyzed the reduction of H_2_O_2_ using the Fc-MeOH as the electron donor, in situ generating its redox counterpart, that is, [Fc-MeOH]^+^. This cation was reduced back to Fc-MeOH at the electrode surface, and the produced current signal was directly proportional to the concentration of the target sequence in the solution. We refer to our previous publication for more experimental details on the performance of the two-electrode electrochemical cell.^40^ Calibration curves were produced in triplicate in standard hybridization buffer. Thereafter, the study was repeated using solutions of the target RNA sequence in a UTM from Deltalab to simulate the real matrix of collected nasopharyngeal swab samples.

### SARS-CoV-2 RNA Detection in Clinically Relevant Samples

Samples from patients included in this study were provided by the biobank of the Germans Trias i Pujol Research Institute (IGTP), integrated with the Spanish National Biobanks Network, and they were processed following standard operating procedures with the appropriate approval of the Ethics and Scientific Committees. All details about the assays that were carried out with these samples are included in the SI.

Some 100 μL of each sample was required to perform the analysis with the electrochemical device, following the procedure described in the SI.

## Conclusions

In summary, we have developed an analytical platform based on the advantageous combination of paper microfluidics, electrochemical transduction, and enzyme-based hybridization assays performed on magnetic nanoparticles for the rapid, accurate, selective, and sensitive detection of SARS-CoV-2 viral RNA within a time period of less than 40 min. Following the optimization of the magnetoassay parameters, the analytical performance of the electrochemical device was evaluated both in standard hybridization buffer solutions and in a UTM, spiked with different concentrations of the synthetic target oligonucleotide. The performance assessment of the device evidenced the influence of the UTM composition on the analytical performance but without compromising the device application in the molecular detection of SARS-CoV-2 in nasopharyngeal swabs, without the need for any RNA extraction and amplification steps. Sensitivity and specificity values of 100 and 93% were achieved, respectively, in the retrospective study carried out with 34 samples. The quantitative detection of RNA with the electrochemical device showed a lack of correlation with the Ct values provided by the RT-PCR analysis, mainly in samples showing Ct values below 20. The interaction of the capture DNA probe immobilized on the magnetic nanoparticles with the RNA target sequence, located in the nsp13 coding region, may be hindered to some extent by the RNA intricate secondary and 3D structures in that region and by RNA intermolecular interactions. Such effects appeared to be more pronounced at high viral loads. This was ascertained by a controlled cleaving process of the RNA whole molecule using Zn^2+^ ions and by performing a series of dilutions with some representative samples.

The presented analytical platform outperforms other amplification-free approaches and can be of potential use to rapidly detect the Covid-19 disease in its different infection stages. Moreover, it is a highly versatile tool that could easily be adapted to detect other infectious diseases that might also require the setup of effective screening programs at the point of need.

## Supplementary Material



## Notes

Experimental details; scheme of the paper component; schemes of the hybridization assay and reactions; sandwich hybridization assay; dose–response curves resulting from the analyses; WHO target product profile; secondary structure of the SARS-CoV-2 genome; and amplification-free electrochemical approaches (PDF)

## References

[1] Bhalla N., Pan Y., Yang Z., Payam A. F. (2020). Opportunities and challenges for
biosensors and nanoscale analytical tools for pandemics: COVID-19. ACS Nano.

[2] WHO . https://www.who.int/news/item/05-05-2023-statement-on-the-fifteenth-meeting-of-the-international-health-regulations-(2005)-emergency-committee-regarding-the-coronavirus-disease-(covid-19)-pandemic (accessed Feb 07, 2025).

[3] WHO . Coronavirus (COVID-19) Dashboard. https://covid19.who.int/ (accessed Feb 07, 2025).

[4] WHO . Coronavirus (COVID-19) Dashboard. https://covid19.who.int/table (accessed Feb 07, 2025).

[5] WHO . https://www.who.int/docs/default-source/coronaviruse/09082023eg.5_ire_final.pdf?sfvrsn=2aa2daee_3 (accessed Aug 14, 2024).

[6] WHO . https://www.who.int/docs/default-source/coronaviruse/15042024_jn1_ure.pdf?sfvrsn=8bd19a5c_7 (accessed Aug 14, 2024).

[7] WHO . https://www.who.int/publications/i/item/WHO-WHE-SPP-2023.1 (accessed Feb 02, 2025).

[8] de
Araujo W. R., Lukas H., Torres M. D. T., Gao W., de la Fuente-Núñez C. (2024). Low-cost biosensor technologies for
rapid detection of COVID-19 and future pandemics. ACS Nano.

[9] Li Z., Xu X., Wang D., Jiang X. (2023). Recent advancements in nucleic acid
detection with microfluidic chip for molecular diagnostics. TrAC, Trends Anal. Chem..

[10] Parassol B. G., Takeuti N. N. K., Faria H. A. M., Jorge K. C., Sampaio I., Zucolotto V., Vieira N. C. S. (2024). Biosensors for amplification-free
viral RNA detection. Biosens. Bioelectron.:
X.

[11] Seo G., Lee G., Kim M. J., Baek S. H., Choi M., Ku K. B., Lee C. S., Jun S., Park D., Kim H. G., Kim S. J., Lee J. O., Kim B. T., Park E. C., Kim S. (2020). Rapid detection of
COVID-19 causative virus (SARS-CoV-2) in human
nasopharyngeal swab specimens using field-effect transistor-based
biosensor. ACS Nano.

[12] Pokhrel P., Hu C., Mao H. (2020). Detecting
the coronavirus (COVID-19). ACS Sens..

[13] Broughton J. P., Deng X., Yu G., Fasching C. L., Servellita V., Singh J., Miao X., Streithorst J. A., Granados A., Sotomayor-Gonzalez A., Zorn K., Gopez A., Hsu E., Gu W., Miller S., Pan C. Y., Guevara H., Wadford D. A., Chen J. S., Chiu C. Y. (2020). CRISPR–Cas12-based
detection of SARS-CoV-2. Nat. Biotechnol..

[14] Sanjay S. T., Fu G., Dou M., Xu F., Liu R., Qi H., Li X. (2015). Biomarker detection for disease diagnosis using cost-effective microfluidic
platforms. Analyst.

[15] Leonardi A. A., Sciuto E. L., Lo Faro M. J., Fazio B., Rizzo M. G., Calabrese G., Francioso L., Picca R., Nastasi F., Mancuso G., Spinella C., Knoll W., Irrera A., Conoci S. (2023). SARS-CoV-2
and omicron variant detection with a high
selectivity, sensitivity, and low-cost silicon bio-nanosensor. Nano Sel..

[16] Dighe K., Moitra P., Alafeef M., Gunaseelan N., Pan D. (2022). A rapid RNA extraction-free lateral flow assay for molecular point-of-care
detection of SARS-CoV-2 augmented by chemical probes. Biosens. Bioelectron..

[17] Gutiérrez-Gálvez L., del Caño R., Menéndez-Luque I., García-Nieto D., Rodríguez-Peña M., Luna M., Pineda T., Pariente F., García-Mendiola T., Lorenzo E. (2022). Electrochemiluminescent
nanostructured DNA biosensor for SARS-CoV-2 detection. Talanta.

[18] Campos-Ferreira D., Visani V., Córdula C., Nascimento G. A., Montenegro L. M. L., Schindler H. C., Cavalcanti I. M. F. (2021). COVID-19
challenges: From SARS-CoV-2 infection to effective point-of-care diagnosis
by electrochemical biosensing platforms. Biochem.
Eng. J..

[19] Calorenni P., Leonardi A. A., Sciuto E. L., Rizzo M. G., Faro M. J. L., Fazio B., Irrera A., Conoci S. (2023). PCR-free innovative
strategies for SARS-CoV-2 detection. Adv. Healthcare
Mater..

[20] Pohanka M., Skládal P. (2008). Electrochemical biosensors - principles
and applications. J. Appl. Biomed..

[21] Kashefi-Kheyrabadi L., Nguyen H. V., Go A., Lee M. H. (2023). Ultrasensitive and
amplification-free detection of SARS-CoV-2 RNA using an electrochemical
biosensor powered by CRISPR/Cas13a. Bioelectrochemistry.

[22] Damiati S., Sopstad S., Peacock M., Akhtar A. S., Pinto I., Soares R. R. G., Russom A. (2021). Flex printed
circuit board implemented
graphene-based DNA sensor for detection of SARS-CoV-2. IEEE Sens. J..

[23] Dou Y., Su J., Chen S., Li T., Wang L., Ding X., Song S., Fan C. (2022). A smartphone-based
three-in-one biosensor
for co-detection of SARS-CoV-2 viral RNA, antigen and antibody. Chem. Commun..

[24] Gao J., Wang C., Wang C., Chu Y., Wang S., Sun M. Y., Ji H., Gao Y., Wang Y., Han Y., Song F., Liu H., Zhang Y., Han L. (2022). Poly-l-lysine-modified
graphene field-effect transistor biosensors for ultrasensitive breast
cancer miRNAs and SARS-CoV-2 RNA detection. Anal. Chem..

[25] Song Z., Ma Y., Chen M., Ambrosi A., Ding C., Luo X. (2021). Electrochemical
biosensor with enhanced antifouling capability for COVID-19 nucleic
acid detection in complex biological media. Anal. Chem..

[26] Heo W., Lee K., Park S., Hyun K. A., Jung H. (2022). Electrochemical
biosensor
for nucleic acid amplification-free and sensitive detection of severe
acute respiratory syndrome coronavirus 2 (SARS-CoV-2) RNA via CRISPR/Cas13a
trans-cleavage reaction. Biosens. Bioelectron..

[27] Cajigas S., Alzate D., Fernández M., Muskus C., Orozco J. (2022). Electrochemical
genosensor for the specific detection of SARS-CoV-2. Talanta.

[28] Roychoudhury A., Allen R. J., Curk T., Farrell J., McAllister G., Templeton K., Bachmann T. T. (2022). Amplification free
detection of SARS-CoV-2
using multi-valent binding. ACS Sens..

[29] Alafeef M., Dighe K., Moitra P., Pan D. (2020). Rapid, ultrasensitive,
and quantitative detection of SARS-CoV-2 using antisense oligonucleotides
directed electrochemical biosensor chip. ACS
Nano.

[30] Kashefi-Kheyrabadi L., Nguyen H. V., Go A., Baek C., Jang N., Lee J. M., Cho N. H., Min J., Lee M. H. (2022). Rapid,
multiplexed, and nucleic acid amplification-free detection of SARS-CoV-2
RNA using an electrochemical biosensor. Biosens.
Bioelectron..

[31] Ji D., Guo M., Wu Y., Liu W., Luo S., Wang X., Kang H., Chen Y., Dai C., Kong D., Ma H., Liu Y., Wei D. (2022). Electrochemical detection of a few
copies of unamplified SARS-CoV-2 nucleic acids by a self-actuated
molecular system. J. Am. Chem. Soc..

[32] Lomae A., Preechakasedkit P., Hanpanich O., Ozer T., Henry C. S., Maruyama A., Pasomsub E., Phuphuakrat A., Rengpipat S., Vilaivan T., Chailapakul O., Ruecha N., Ngamrojanavanich N. (2023). Label free
electrochemical DNA biosensor
for COVID-19 diagnosis. Talanta.

[33] del
Caño R., García-Mendiola T., García-Nieto D., Álvaro R., Luna M., Iniesta H. A., Coloma R., Diaz C. R., Milán-Rois P., Castellanos M., Abreu M., Cantón R., Galán J. C., Pineda T., Pariente F., Miranda R., Somoza Á., Lorenzo E. (2022). Amplification-free detection of SARS-CoV-2 using gold
nanotriangles functionalized with oligonucleotides. Microchim. Acta.

[34] Zhao H., Liu F., Xie W., Zhou T. C., OuYang J., Jin L., Li H., Zhao C. Y., Zhang L., Wei J., Zhang Y. P., Li C. P. (2021). Ultrasensitive supersandwich-type electrochemical sensor for SARS-CoV-2
from the infected COVID-19 patients using a smartphone. Sens. Actuators, B.

[35] Wignarajah S., Suaifan G. A. R. Y., Bizzarro S., Bikker F. J., Kaman W. E., Zourob M. (2015). Colorimetric assay for the detection
of typical biomarkers
for periodontitis using a magnetic nanoparticle biosensor. Anal. Chem..

[36] Kaneta T., Alahmad W., Varanusupakul P. (2019). Microfluidic
paper-based analytical
devices with instrument-free detection and miniaturized portable detectors. Appl. Spectrosc. Rev..

[37] Gutiérrez-Capitán M., Baldi A., Fernández-Sánchez C. (2020). Electrochemical
paper-based biosensor devices for rapid detection of biomarkers. Sensors.

[38] Rezvani
Jalal N., Mehrbod P., Shojaei S., Labouta H. I., Mokarram P., Afkhami A., Madrakian T., Los M. J., Schaafsma D., Giersig M., Ahmadi M., Ghavami S. (2021). Magnetic nanomaterials in microfluidic sensors for
virus detection: a review. ACS Appl. Nano Mater..

[39] Tymm C., Zhou J., Tadimety A., Burklund A., Zhang J. X. J. (2020). Scalable
COVID-19 detection enabled by lab-on-chip biosensors. Cell. Mol. Bioeng..

[40] Gutiérrez-Capitán M., Baldi A., Merlos Á., Fernández-Sánchez C. (2022). Array of individually
addressable two-electrode electrochemical cells sharing a single counter/reference
electrode for multiplexed enzyme activity measurements. Biosens. Bioelectron..

[41] Gutiérrez-Capitán M., Sanchís A., Carvalho E. O., Baldi A., Vilaplana L., Cardoso V. F., Calleja Á., Wei M., de la Rica R., Hoyo J., Bassegoda A., Tzanov T., Marco M.-P., Lanceros-Méndez S., Fernández-Sánchez C. (2023). Engineering
a point-of-care paper-microfluidic electrochemical device applied
to the multiplexed quantitative detection of biomarkers in sputum. ACS Sens..

[42] Ayres L. B., Pimentel G. J. C., Costa J. N. Y., Piazzetta M. H. O., Gobbi A. L., Shimizu F. M., Garcia C. D., Lima R. S. (2024). Ultradense
array of on-chip sensors for high-throughput electrochemical analyses. ACS Sens..

[43] Bezinge L., Tappauf N., Richards D. A., Shih C. J., deMello A. J. (2023). Rapid electrochemical
flow analysis of urinary creatinine on paper: unleashing the potential
of two-electrode detection. ACS Sens..

[44] Bezinge L., Shih C. J., Richards D. A., deMello A. J. (2024). Electrochemical
paper-based microfluidics: harnessing capillary flow for advanced
diagnostics. Small.

[45] González
del Campo M. M., Vaquer A., de la Rica R. (2022). Polymer components
for paper-based analytical devices. Adv. Mater.
Technol..

[46] Trinh K. T. L., Chae W. R., Lee N. Y. (2022). Recent
advances in the fabrication
strategies of paper-based microfluidic devices for rapid detection
of bacteria and viruses. Microchem. J..

[47] Aviñó A., Cuestas-Ayllón C., Gutiérrez-Capitán M., Vilaplana L., Grazu V., Noé V., Balada E., Baldi A., Félix A. J., Aubets E., Valiuska S., Domínguez A., Gargallo R., Eritja R., Marco M.-P., Fernández-Sánchez C., Martínez de la Fuente J., Ciudad C. J. (2022). Detection of SARS-CoV-2
virus by triplex enhanced nucleic acid detection assay (TENADA). Int. J. Mol. Sci..

[48] European Centre for Disease Prevention and Control . https://www.ecdc.europa.eu/en/covid-19/variants-concern (accessed Jan 20, 2024).

[49] Ciudad C.
J., Valiuska S., Rojas J. M., Nogales-Altozano P., Aviñó A., Eritja R., Chillón M., Sevilla N., Noé V. (2024). Polypurine
Reverse Hoogsteen hairpins
as a therapeutic tool for SARS-CoV-2 infection. J. Biol. Chem..

[50] Wiraswati H. L., Gaffar S., Ekawardhani S., Fauziah N., Rinawan F. R., Widyatmoko L., Laelalugina A., Arimdayu A. R., Kusniati T., Andari C. D., Faridah L. (2022). Evaluation and clinical validation
of guanidine-based inactivation transport medium for preservation
of SARS-CoV-2. Adv. Pharmacol. Pharm. Sci..

[51] Baek Y. H., Park M. Y., Lim H. J., Jung H. S., Yang J. H., Sohn Y. H., Lee S. H., Park J. E., Yang Y. J. (2022). Evaluation
of alternative transport media for RT-qPCR-based SARS-CoV-2 testing. Int. J. Anal. Chem..

[52] Mukae K., Takei O., Imai F., Kamijo T. (2023). Development of RNA/DNA
automated extraction and purification device for infectious disease
diagnosis. Pract. Lab. Med..

[53] Sabat J., Subhadra S., Rath S., Ho L. M., Kanungo S., Panda S., Mandal M. C., Dash S., Pati S., Turuk J. (2021). Yielding quality viral
RNA by using two different chemistries: a
comparative performance study. Biotechniques.

[54] World Health Organization - R&D Blueprint . COVID-19 Target Product Profiles for Priority Diagnostics to Support Response to the COVID-19 Pandemic; R&D Blueprint. https://www.who.int/publications/m/item/covid-19-target-product-profiles-for-priority-diagnostics-to-support-response-to-the-covid-19-pandemic-v.0.1 (accessed March 16, 2025).

[55] Mabey D., Peeling R. W., Ustianowski A., Perkins M. D. (2004). Diagnostics for
the developing world. Nat. Rev. Microbiol..

[56] Land K. J., Boeras D. I., Chen X. S., Ramsay A. R., Peeling R. W. (2019). REASSURED
diagnostics to inform disease control strategies, strengthen health
systems and improve patient outcomes. Nat. Microbiol..

[57] Baldeh M., Bawa F. K., Bawah F. U., Chamai M., Dzabeng F., Jebreel W. M. A., Kabuya J. B. B., Molemodile Dele-Olowu S. K., Odoyo E., Rakotomalala Robinson D., Cunnington A. J. (2024). Lessons
from the pandemic: new best practices in selecting molecular diagnostics
for point-of-care testing of infectious diseases in sub-Saharan Africa. Expert Rev. Mol. Diagn..

[58] Fernández-Sánchez, C. ; Gutiérrez-Capitán, M. ; Baldi, A. Biosensor System for Multiplexed Detection of Biomarkers. EP20382721.7 2022.

[59] Najjar D., Rainbow J., Sharma Timilsina S., Jolly P., de Puig H., Yafia M., Durr N., Sallum H., Alter G., Li J. Z., Yu X. G., Walt D. R., Paradiso J. A., Estrela P., Collins J. J., Ingber D. E. (2022). A lab-on-a-chip
for the concurrent electrochemical detection of SARS-CoV-2 RNA and
anti-SARS-CoV-2 antibodies in saliva and plasma. Nat. Biomed. Eng..

[60] ThermoFisherScientific, Inc. RNA Fragmentation Reagents. https://www.thermofisher.com/document-connect/document-connect.html?url=https://assets.thermofisher.com/TFS-Assets%2FLSG%2Fmanuals%2Fsp_8740.pdf (accessed June 13, 2023).

[61] Rangan R., Watkins A. M., Chacon J., Kretsch R., Kladwang W., Zheludev I. N., Townley J., Rynge M., Thain G., Das R. (2021). De novo 3D models of
SARS-CoV-2 RNA elements from consensus experimental
secondary structures. Nucleic Acids Res..

[62] Cao C., Cai Z., Xiao X., Rao J., Chen J., Hu N., Yang M., Xing X., Wang Y., Li M., Zhou B., Wang X., Wang J., Xue Y. (2021). The architecture
of the SARS-CoV-2 RNA genome inside virion. Nat. Commun..

[63] Chakraborty C., Bhattacharya M., Sharma A. R., Chatterjee S., Agoramoorthy G., Lee S. S. (2024). Structural landscape of nsp coding
genomic regions of SARS-CoV-2-ssRNA genome: a structural genomics
approach toward identification of druggable genome, ligand-binding
pockets, and structure-based druggability. Mol.
Biotechnol..

[64] Aubets E., Chillon M., Ciudad C. J., Noé V. (2021). PolyPurine
Reverse Hoogsteen hairpins work as RNA species for gene silencing. Int. J. Mol. Sci..

[65] Noé V., Aubets E., Félix A. J., Ciudad C. J. (2021). Nucleic acids therapeutics
using PolyPurine Reverse Hoogsteen hairpins. Biochem. Pharmacol..

[66] Domínguez A., Gargallo R., Cuestas-Ayllón C., Grazu V., Fàbrega C., Valiuska S., Noé V., Ciudad C. J., Calderón E. J., de la Fuente J. M., Eritja R., Aviñó A. (2024). Biophysical evaluation of antiparallel
triplexes for biosensing and biomedical applications. Int. J. Biol. Macromol..

[67] Orozco J., Suárez G., Fernández-Sánchez C., McNeil C., Jiménez-Jorquera C. (2007). Characterization
of
ultramicroelectrode arrays combining electrochemical techniques and
optical microscopy imaging. Electrochim. Acta.

[68] Chemicell GmbH . Protocol A10. http://www.chemicell.com/products/protocols/docs/fluidMAG-ARA.pdf (accessed June 13, 2023).

[69] CellMosaic Inc. HRP-Oligo Conjugation Kit (Thiol Oligo) User Reference Guide. https://www.cellmosaic.com/content/Manual/DCM53402_HRP_Oligo_Kit_VB.pdf (accessed April 21, 2023).

