# Supplementary material for: Unraveling the Amplification-Free Quantitative Detection of Viral RNA in Nasopharyngeal Swab Samples Using a Compact Electrochemical Rapid Test Device
Source: Anal Chem. 2025 May 30;97(22):11863–73. doi: 10.1021/acs.analchem.5c01605 (PMC12163870; doi:10.1021/acs.analchem.5c01605)
Supplement: Supplementary file 1 [file ac5c01605_si_001.pdf]

Supporting Information

Unravelling the amplification-free quantitative detection of viral RNA in nasopharyngeal swab samples using a compact electrochemical rapid test device

Manuel Gutiérrez-Capitán[a], Eva Balada[b,c], Anna Aviñó[b,c], Lluïsa Vilaplana[b,c], Roger Galve[b,c], Alícia Lacoma[d,e], Antonio Baldi[a], Antonio Alcamí[f], Véronique Noé[g,h], Carlos J. Ciudad[g,h], Ramón Eritja[b,c], María-Pilar Marco[b,c], César Fernández-Sánchez[a,c*]

[a] Instituto de Microelectrónica de Barcelona (IMB-CNM), CSIC, 08193 Bellaterra, Spain

[b] Institute for Advanced Chemistry of Catalonia (IQAC), CSIC, 08034 Barcelona, Spain

[c] Centro de Investigación Biomédica en Red de Bioingeniería, Biomateriales y Nanomedicina (CIBER-BBN), Instituto Carlos III, 28029 Madrid, Spain

[d] Institut d'Investigació Germans Trias i Pujol (IGTP), Camí de les Escoles, 08916 Badalona, Spain.

[e] CIBER Enfermedades Respiratorias (CIBERES), Instituto de Salud Carlos III, 28029 Madrid, Spain

[f] Centro de Biología Molecular Severo Ochoa, CSIC, and Universidad Autónoma de Madrid, Madrid, 28049, Spain

[g] Department of Biochemistry and Physiology, School of Pharmacy and Food Sciences, University of Barcelona (UB), 08028 Barcelona, Spain

[h] Instituto de Nanociencia y Nanotecnología (IN2UB), University of Barcelona (UB), 08028 Barcelona, Spain

*Email: cesar.fernandez@csic.es

Table of Contents

Experimental details ……………………………………………………………………....S3

Figure S1. Scheme of the paper component ……………………………………………..S8

Figure S2. Scheme of the hybridization assay and reactions ………………………….....S9

Sandwich hybridization assay (Figure S3) ..……..……………………………………...S10

Table S1. WHO target product profile …………………………………………………..S12

Figure S4. Secondary structure of the SARS-CoV-2 genome ……………………….....S15

Table S2. Amplification-free electrochemical approaches ..…………………………….S17

References ………………………………………………………………………………S18

Experimental details

Reagents and solutions

200 nm diameter carboxylated magnetic nanoparticles (MNPs, fluidMAG-ARA, Chemicell GmbH, Berlin, Germany), 1-ethyl-3-(3-dimethylaminopropyl)carbodiimide hydrochloride (EDC), 0.1 M 2-(N-morpholino)ethanesulfonate (MES) buffer pH 5.0, 10 mM phosphate-buffered saline (PBS) solution pH 7.4, 50 mM tris(hydroxymethyl)aminomethane (TRIS) buffer pH 7.2, 0.1 M citrate/acetate buffer pH 5.5, ferrocenemethanol redox mediator and H_2O_2 enzyme substrate were used. Samples were collected in a Universal Transport Medium (UTM) purchased from Deltalab S.L. (Rubí, Spain) together with the collector swab. A GeneAll buffer containing an inactivating agent was added to all samples.

Electrochemical cell and paper fluidic component

Chips were diced from the wafer, and then cleaned in a cold acid piranha solution for 30 min to remove any organics from the gold electrode surface and kept vacuum-sealed in plastic pouches, until use. A detailed description of the analytical characterization of this two-electrode cell arrangement has previously been reported.[1] It also shows that this electrochemical cell configuration is suited for the electrochemical detection of HRP enzyme label in electrochemical magneto-immunoassays, by performing the corresponding enzymatic reaction in the presence of ferrocenemethanol (Fc-MeOH) mediator.[2]

The paper component consisted of 25 mm long fluidic structures. These included a 2-mm wide fluidic area connected to a 4-mm diameter circular sample addition area at one end and a 1-mm wide fluidic channel at the other end, designed with the purpose of a more controlled liquid flow. A 5×50 mm^2 absorbent pad, which was folded and placed overlapping by 2.5 mm the front and back faces of the fluidic structure, was also defined. Both paper structures were made of Whatman cellulose chromatography paper, Grade 1 (Cytiva, Marlborough, MA, USA). Two polyvinyl layers (150-µm-thick sign vinyl film from Metamark Ld., Lancaster, UK) were also designed. The paper structures were aligned and sandwiched in between them. The vinyl top layer included two open windows for exposing the sample addition area, and the sink area to enable the evaporation of the liquids arriving to the absorbent pad. The vinyl bottom layer defined a third open window to expose the detection

area of the paper channel and put it in contact with the electrochemical cell. On both vinyl layers, additional windows were opened to get access to the connector pads of the chip. Four 1.5-mm diameter hollow circles were also defined at the corners to facilitate the alignment of the paper component on top of the electrochemical chip. **Figure S1** above shows a detailed scheme of these parts.

Cartridge assembly

The bottom part of the cartridge defined an area where the electrochemical cell was placed. Also, it included a 2-mm diameter Neodymium magnet (Supermagnete, Gottmadingen, Germany), placed at a 1.5-mm distance from the WE. In addition, four 1.5-mm diameter poles were included where the 4 vinyl hollow circles mentioned above fit to easily align the paper component. On the other hand, three windows were opened on the cartridge lid to leave the sample addition and the sink areas of the paper component uncovered and to insert a three-pin spring-loaded connector (Preci-dip SA, Delémont, Switzerland) for connecting the pads of the electrochemical cell. A fourth window was opened on the lid just above the detection area, where a 3-mm wide flexible bridge was machined on a 150-µm thick PMMA substrate. This flexible structure also incorporated a 150 µm thick polyvinyl pad to push down the paper channel and thus ensure the intimate contact between the paper and the electrodes. In order to keep a constant pressure between them, four clamping structures were used. This approach allows the ease assembling and disassembling of the cartridge to facilitate the replacement of the paper component after one measurement. The overall dimensions of the thus fabricated device were 33×67×19 mm^3.

Optimization of the magneto-assay

Firstly, 83.3 µg/mL and 166.7 µg/mL MNPs dilutions were prepared in TRIS buffer containing 1 mM EDTA and 1 M NaCl (hybridization buffer). To 300 µL of these suspensions, 100 µL of standard solutions were added in different 1.5 mL tubes. These standard solutions contained increasing concentrations of the target DNA sequence (0, 0.01, 0.065, 0.41, 2.56 and 16 nM). The mixture was incubated for 15 min at RT at 750 rpm. Then, 100 µL of 83.3 nM HRP-conjugated reporter sequence (HRP-RS) solution were added and incubated at 750 rpm for 5 more min at RT. Once the hybridization reactions took place, the MNPs were trapped with the aid of an external magnet, washed three times with 1 mL of

PBS solution containing 0.05% Tween 20 (PBST) and 100 µL of TMB liquid substrate for ELISA (T4444 from Sigma-Aldrich) was added and incubated at 750 rpm for 5 min at RT. Then, the supernatants were transferred to microplate wells and the absorbance was read at 650 nm with a SpectramaxPlus microplate spectrophotometer controlled by SoftmaxPro v4.7 software (Molecular Devices, Sunnyvale, CA, USA). With these signal values, dose-response curves were constructed for each functionalized MNP dilution.

Once the concentration of MNPs was set, the effect of the concentration of the HRP- reporter sequence conjugate (HRP-RS) on the analytical signal was studied by repeating the same assay performed with three different concentrations of this probe (250, 83.3 and 41.6 nM). Finally, dose response curves were performed in triplicate by carrying out the simultaneous incubation of the functionalized MNPs and the HRP-reporter sequence conjugate in solutions containing increasing concentrations of the target sequence for 15 min at RT.

Analytical steps performed for each measurement

1. Target sequence capturing. The hybridization reactions required for the specific detection of the target oligonucleotide were performed in 1.5-mL tubes using the conditions optimized for the magneto-assay, as described above. After the incubation process, the MNPs were collected with a magnet. The reaction solution was discarded and the MNPs were resuspended and concentrated in 100 µL PBST.

2. MNP flowing and trapping on the device. Once the sample/standard solution was pretreated, some 7 µL of the MNP concentrated suspension was cast on the sample addition area of the fluidic channel in the electrochemical biosensor device and allowed to flow by capillary action. Solution took around 3 min to reach the area over the cartridge-inserted magnet where the MNPs were trapped. Then, a washing step was carried out by adding 7 µL citrate/acetate buffer solution, which was left to flow until the added solution could not be seen at the sample addition area, taking around 6 min.

3. Enzymatic reaction and electrochemical measurement. Finally, 7 µL of citrate/acetate buffer pH 5.5 containing 1.7 mM H_2O_2 and 2 mM Fc-MeOH was added to the fluidic channel, and it was left to flow for 5 min. At this time, a chronoamperometric measurement at −0.15 V vs. Au CRE was carried out. This potential set was chosen from cyclic voltammetric experiments performed in a previous work.[2] The measurement lasted 3 s, the current intensity being recorded every 200 ms. The current value at 1.6 s was used as the analytical signal.

Functionalization of MNPs and thiol-reporter sequence conjugation

Some 5 mg of MNPs were washed 2×1 mL in MES buffer by using a magnetic separator (MagnetoPURE, Chemicell GmbH) and incubated with 5 mg of EDC in 250 µL of MES buffer at 750 rpm for 10 min at room temperature (RT) using a circular shaker (MS 3 digital, IKA GmbH, Staufen, Germany). The MNPs were then washed 2×1 mL with MES buffer and resuspended in 125 µL of MES buffer. 0.17 nmols of the capture probe were immediately added and incubated for 2 h at RT at 750 rpm. Then, the MNPs were again washed 3×1 mL PBS followed by a blocking step in PBS containing 0.1% BSA for 2 h at RT at 750 rpm. Finally, the MNPs were collected, the solution was removed and the resulting modified MNPs were resuspended in 1 mL PBS containing 0.05% sodium azide, used as preservative. Some 200 µL aliquots were prepared and stored in the fridge at 4 ºC, until use.

The labelling of the reported sequence with HRP was as follows. An amount of 5 nmol of disulphide-oligonucleotide was reduced by incubation at 37 ºC for 30 min in an incubator set (ThermoMixer F1.5, Eppendorf, Hamburg, Germany). Once reduced, the thiol-oligonucleotide detection probe was incubated with the activated maleimide-HRP overnight in the dark at room temperature (RT). After a purification step to remove the excess of HRP using an ion exchange column, the HRP reporter probe conjugate was eluted to an approximate concentration of 6 µM in 400 µL of 50 mM TRIS buffer pH 8.0 containing 1 M NaCl. Some 100 µL aliquots were prepared and stored in the freezer at -20 ºC, until use.

SARS-CoV-2 RNA detection in clinically relevant samples.

A total of 58 samples come from nasopharyngeal swabs were collected from patients during the first two years of the pandemic situation (from September 2020 to February 2022). The UTM from Deltalab was used for the collection, to which a lysis VL buffer from a RNA extraction kit (Riboshpin vRD from GeneAll, Seoul, South Korea) was added to a 1:1 v:v ratio. Then, the samples were split into two groups. The first group, formed by 20 positive and 14 negative samples confirmed by PCR, was collected and directly frozen. The second one, formed by the remaining 16 positive and 8 negative samples confirmed by PCR, included those where the RNA was extracted and then frozen. Repeated freezing and thawing

steps were avoided, though samples might have suffered from these processes to a certain extent during transport from the biobank to the lab. In all cases, once received, samples were kept at −80 ◦C to preserve the viral RNA, until use.

RNA Fragmentation kit based on zinc acetate (AM8740 from Thermo Fisher Scientific, Madrid, Spain)[3] was used with some of the collected samples. This kit cleaves the viral RNA genome into strands with sizes between 60-200 nucleotides. The quantity of RNA for which the kit was recommended (2-20 μg) and the much lower quantity of RNA that could be found in the analyzed clinical samples required the dilution of the Zn acetate reagent before use. A stock of viral RNA was obtained from infected Vero cell cultures provided by the Centro de Biología Molecular Severo Ochoa (CBMSO-CSIC-UAM, Madrid, Spain).

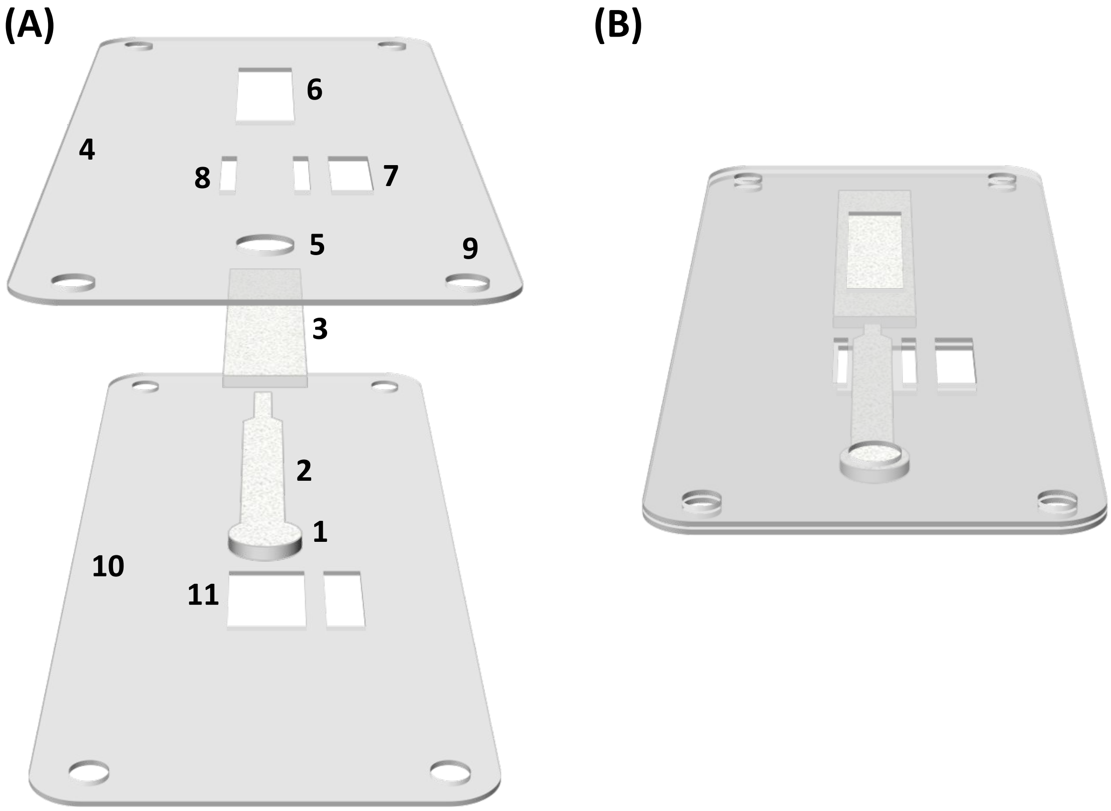

Figure S1. Scheme of the paper component. Overall dimensions: 57×23 mm^2. (A) Exploded view showing the different parts and details of its architecture. 1- sample addition area; 2- paper fluidic channel; 3 – sink / absorbent pad; 4 – top vinyl layer showing open windows for, 5 - sample addition, 6 – fluid evaporation, 7- for accessing the contact pads of the electrochemical transducer by the spring-loaded connectors, 8 – for alignment of the paper channel, 9 - holes for alignment in the cartridge; 10 – bottom vinyl layer showing an additional open window for, 11 - contacting the paper component and the electrochemical transducer. (B) Paper device already mounted.

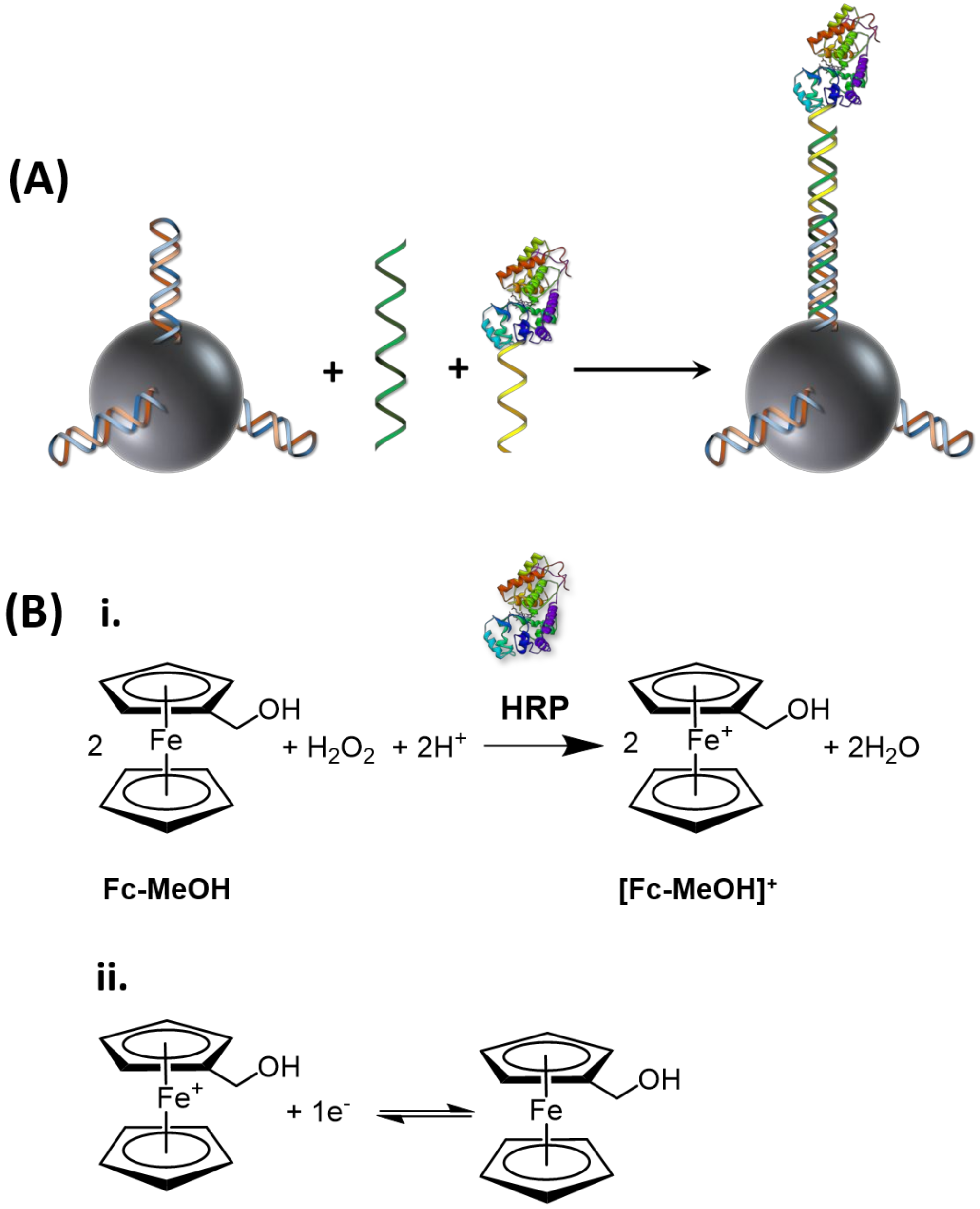

Figure S2. (A) Scheme of the hybridization assay taking place on the MNPs. (B) Enzymatic and electrochemical reactions using Fe-MeOH redox mediator

Sandwich hybridization assay

Figure S3 shows the results of the univariate analysis carried out to set the concentration of the magnetic nanoparticles functionalized with PPRH capture probe (MNP-PPRH) and the HRP-reporter sequence conjugate (HRP-RS). Absorbance measurements were performed using colourless 3,3',5,5'-Tetramethylbenzidine (TMB) as the redox mediator and measuring the absorbance of the blue TMB^{+} produced during the enzymatic reaction at 650 nm. Dose-response curves were recorded in a DNA target sequence concentration ranging from 0.01 to 10 nM. Data was fitted to sigmoidal functions in the semilogarithmic scale. Two different concentrations of the MNP-PPRH were assessed, that is 83.3 and 166.7 µg/mL. The latter provided enhanced analytical signals. However, the former was set for the next experiments because the low background signals recorded gave rise to enhanced signal to background ratios. The second study was performed with three different concentrations of the HRP-RS, the higher absorbance values were again obtained for the highest tested concentration of 250 nM, but the background signal was also high. Regarding the other two concentrations of 83.3 nM and 41.6 nM, both of them provided significantly lower background signals but the former gave rise to a higher sensitivity, clearly shown in the corresponding dose-response curve. Therefore 83.3 µg/mL and 83.3 mM concentrations of MNP-PPRH and HRP-RS were respectively chosen for further studies.

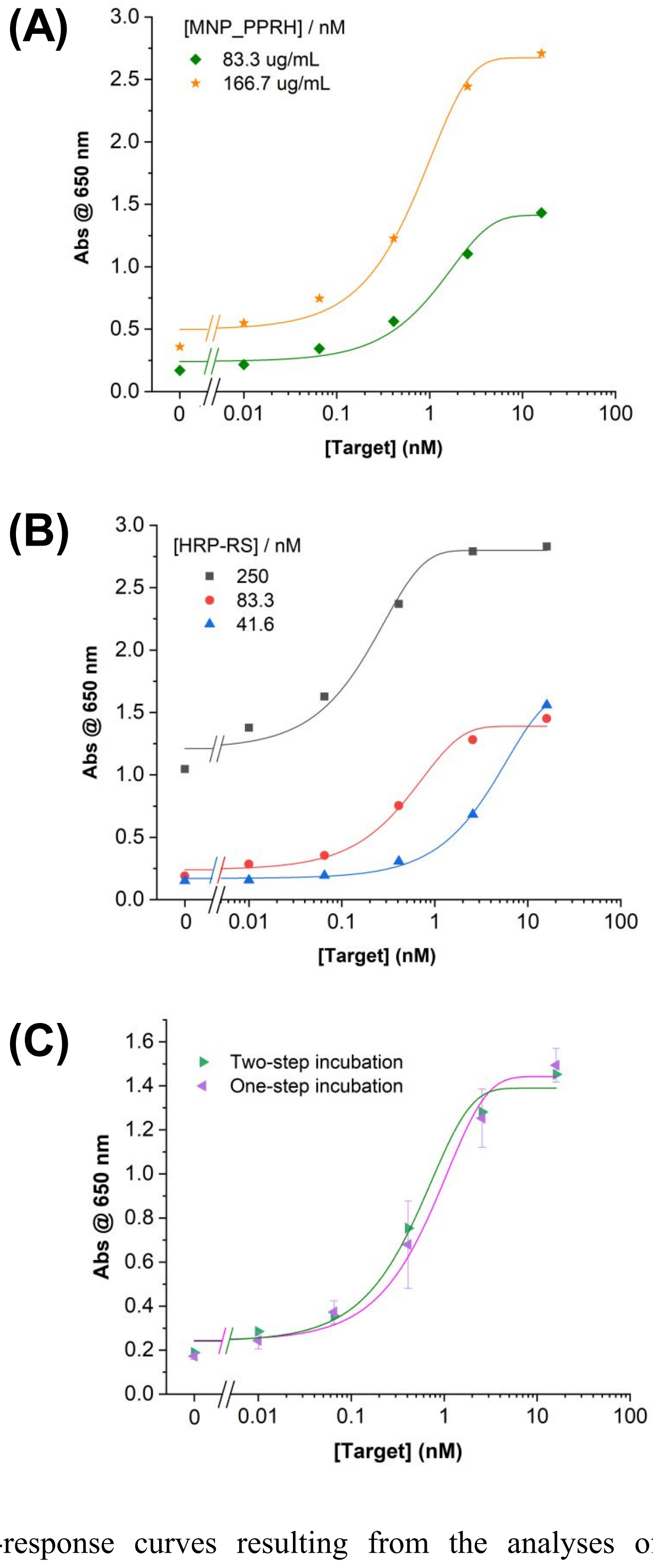

Figure S3. Dose-response curves resulting from the analyses of, (A) two different concentrations of MNP modified with the capture probe (MNP-PPRH), (B) three different concentrations of the HRP-conjugated reporter sequence (HRP-RS), (C) two-step and one-step incubation processes of target sequence and HRP-RS.

Table S1. Target product profile that a point-of-care test should potentially fulfil when applied in suspected COVID-19 cases to diagnose acute viral infection in areas where reference assay testing is not available or turnaround times obviate the test clinical utility.[1]

KEY FEATURE	ACCEPTABLE	DESIRABLE
**Intended Use / Target population**	For screening areas with confirmed SARS-CoV-2 community wide transmission or confirmed outbreaks in closed or semi-closed communities and in high risk groups. In suspected SARS-CoV-2 outbreak situations / to carry out the test in patients with acute or subacute respiratory symptoms or fever or suspicious symptoms and contacts with confirmed or probable Covid-19 patients or living in a community where transmission is very high.<br>For monitoring trends in disease incidence.<br>For slowing and stopping transmission by timely detection of the most infectious cases / to screen population where prevalence is high.	
**Setting**	Outside laboratories including routine and ad-hoc triage/screening points of health care facilities such as emergency units, mobile units and in the community (contact tracing) by health care workers or laboratory technicians with appropriate training in sample collection, biosafety and in the use of the test.	Same as acceptable but can be self-administered and/or performed by trained lay workers (volunteer/community health workers).
**Target molecule**	Molecular: Specific sequences of viral RNA (SARS-CoV-2) or of other pathogens should the device be implemented for the rapid detection of other infectious diseases.<br>Protein: Antigen tests	The same as acceptable as the target analytes for the detection of an infection should be clearly defined.
**Type of analysis**	Qualitative, semi-quantitative or quantitative	Quantitative[2]
**Number of steps to be performed (use of different reagents / incubation steps)**	≤ 3	1, with the potential for digitally guided workflows and built-in timers to reduce user errors on timed steps

**Time from sample collection to result**	≤ 40 min	≤ 20 min
**Limit of detection**	Equivalent to 10^6 genomic copies/mL or Ct ≈ 25-30. The establishment of a limit of detection is crucial and timely quantitative results highly demanded since viral loads in patient specimens and associated infectivity is critical to anticipate viral transmission and effectively tackle it. A point-of-care device that can consistently detect the most infectious patients (e.g. LOD 10^6) are required in order to interrupt transmission.	Equivalent to 10^4 genomic copies/mL or Ct≈>30
**Sensitivity**	≥ 80%	≥90%
**Specificity**	≥ 97%	>99%
**Interpretation**	Visual manual and / or hardware reader	Visual manual read or digital readout via smartphone application reader with connectivity
**Sample type**	Nasopharyngeal, oropharyngeal swab, nasal swab, nasal wash, sputum	Anterior nares, saliva/oral fluid, sputum
**Sample collection device**	Compatible with an existing swab	Compatible with multiple swab materials or none
**End-user**	Training staff in healthcare facilities	Training staff in healthcare facilities or community level or self-administered
**Training needs**	Sample collection, test procedure, results reading and quality control and biosafety (common to all diagnostic tests for infectious diseases) that will take 0.5 days	Sample collection, test procedure, results reading and quality and biosafety (common to all diagnostic tests for infectious diseases) – quick reference guides to be instructed in 2 h
**Sample preparation steps**	1	0

**Sample minimum volume**	Single swab and minimal extraction buffer / diluent.	Single swab and minimal extraction buffer / diluent.
**Operating conditions**	Operating conditions 15-35°C; 25-80% relative humidity up to 1,500m.	10-40°C; 25-90% relative humidity up to 3,000m.<br>Ideally, tests could support conditions in tropical countries.
**Quality control**	Internal control for assessing correct device performance integrated in the device; positive and negative control, calibration control for reader, if applicable.	Internal control for assessing correct device performance integrated in the device; positive and negative control, calibration control for reader, if applicable.
**Remote connectivity and data storage capacity**	Not required for reader independent tests; if device based – remote export of data possible.	Test compatible with readers and other data capture devices; internal memory for storage as well.
**Need for additional equipment**	Handheld battery or solar powered with more than 8 h of use without recharging	No additional equipment but, if required it should have potential digital connectivity through a smartphone app.

[1]Adapted with permission from "Target product profiles for priority diagnostics to support response to the COVID-19 pandemic v.1.0," 2020. This adaptation was not created by WHO. WHO is not responsible for the content or accuracy of this adaptation. The original edition shall be the binding and authentic edition". URL - https://www.who.int/publications/m/item/covid-19-target-product-profiles-for-priority-diagnostics-to-support-response-to-the-covid-19-pandemic-v.0.1
[2]Quantitative may be desirable for more effective patient management in emergency units and other clinical environments

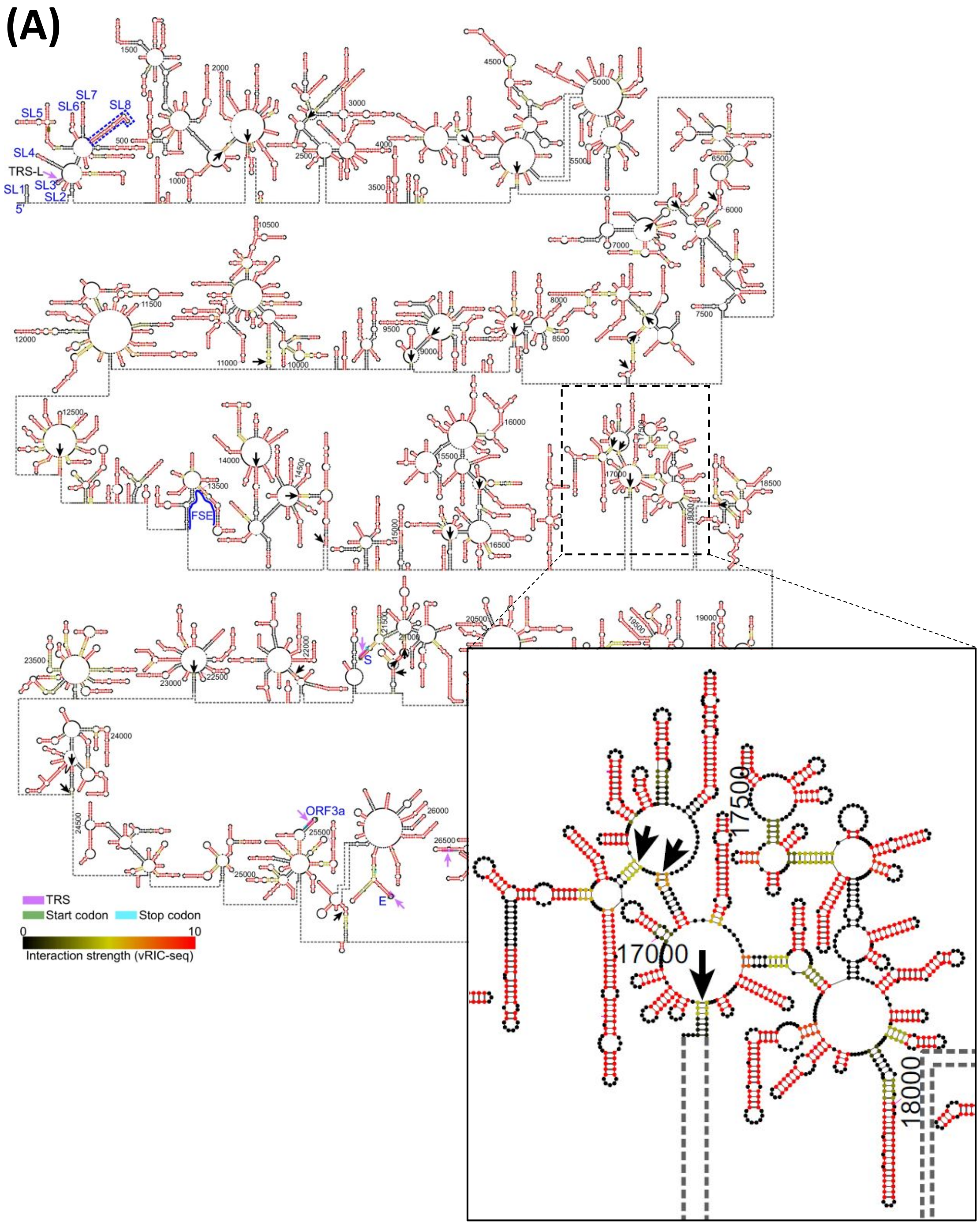

Figure S4

(continued)

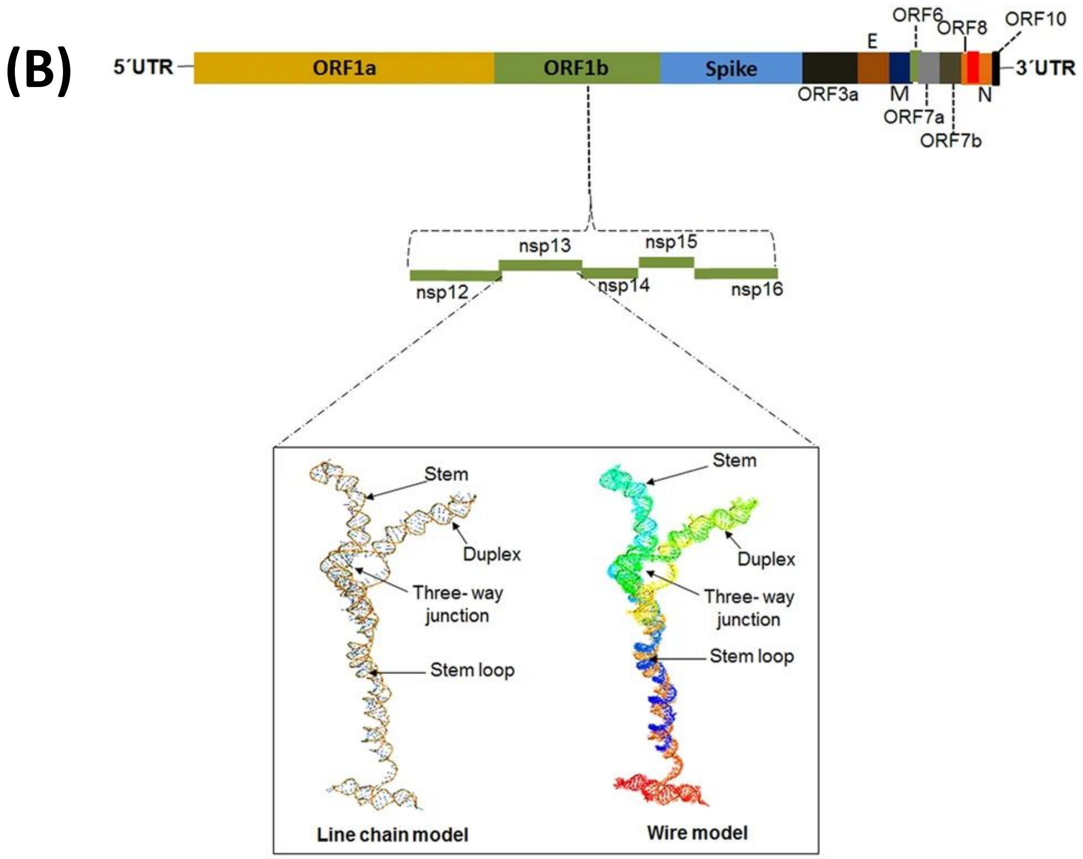

Figure S4. (A) Secondary structure of the SARS-CoV-2 genome. Adapted from Cao, C. *et al.* The Architecture of the SARS-CoV-2 RNA Genome inside Virion. Nature Communications, 12, 1-14, 2021, Springer Nature. (**B)** 3D model of the RNA of nsp 13 coding region. Adapted with permission from Chakraborty, C. *et al.* Structural Landscape of Nsp Coding Genomic Regions of SARS-CoV-2-SsRNA Genome: A Structural Genomics Approach Toward Identification of Druggable Genome, Ligand-Binding Pockets, and Structure-Based Druggability. Molecular Biotechnology, 66, 641–662, 2024, Springer Nature.

Table S2. Amplification-free electrochemical approaches validated with real clinical samples.

Electrochemical technique[a]	Clinical samples	Pretreatment	Time (min)[b]	S (%)[c]	Sp. (%)[d]	Quantitative	Ref.
Potentiometry	48 nasopharyngeal swabs	Heating at 65 ºC for 30 min and RNA extraction	5	100	100	YES	[4]
DPV	88 clinical specimens (17 sputum, 20 throat swab, 15 urine, 11 faeces, 10 plasma, 8 serum, 3 whole blood, 3 oral swab, 1 saliva)	Heating at 56 ºC for 30 min and RNA extraction	180	85.5	46.2	NO	[5]
SWV	21 nasopharyngeal swab	RNA extraction	60	100	100	YES	[6]
SWV and DPV	14 nasopharyngeal swab	Heating at 56 ºC for 30 min	31	100	100	NO	[7]
Amperometry	10 nasopharyngeal swab	RNA extraction	42	100	100	YES	[8]
Chronoamperometry	34 nasopharyngeal swab	No	40	100	93	YES	This work

[a] DPV: differential pulse voltammetry; SWV: squared-wave voltammetry; [b] Total time of analysis including the incubation and other treatment steps previous to the measurement; [c] Sensitivity - Positive samples correctly detected as positive; [d] Specificity - Negative samples correctly detected as negative

References

(1) Gutiérrez-Capitán, M.; Baldi, A.; Merlos, Á.; Fernández-Sánchez, C. Array of individually addressable two-electrode electrochemical cells sharing a single counter/reference electrode for multiplexed enzyme activity measurements. *Biosens. Bioelectron.* **2022**, *201*, 113952.

(2) Gutiérrez-Capitán, M.; Sanchís, A.; Carvalho, E.O.; Baldi, A.; Vilaplana, L.; Cardoso, V.F.; Calleja, Á.; Wei, M.; de la Rica, R.; Hoyo, J.; Bassegoda, A.; Tzanov, T.; Marco, M.-P.; Lanceros-Méndez, S.; Fernández-Sánchez, C. Engineering a point-of-care paper-microfluidic electrochemical device applied to the multiplexed quantitative detection of bi-omarkers in sputum. *ACS Sensors* **2023**, *8* (8), 3032-3042.

(3) ThermoFisherScientific, Inc. RNA Fragmentation Reagents. https://www.thermofisher.com/document-connect/document-connect.html?url=https://assets.thermofisher.com/TFS-Assets%2FLSG%2Fmanuals%2Fsp_8740.pdf (accessed 2023-06-13).

(4) Alafeef, M.; Dighe, K.; Moitra, P.; Pan, D. Rapid, ultrasensitive, and quantitative detection of SARS-CoV-2 using antisense oligonucleotides directed electrochemical biosensor chip. *ACS Nano* **2020**, *14* (12), 17028-17045.

(5) Zhao, H.; Liu, F.; Xie, W.; Zhou, T.C.; OuYang, J.; Jin, L.; Li, H.; Zhao, C.Y.; Zhang, L.; Wei, J.; Zhang, Y.P.; Li, C.P. Ultrasensitive supersandwich-type electrochemical sensor for SARS-CoV-2 from the infected COVID-19 patients using a smartphone. *Sens. Actuators B Chem.* **2021**, *327*, 128899.

(6) Kashefi-Kheyrabadi, L.; Nguyen, H.V.; Go, A.; Baek, C.; Jang, N.; Lee, J.M.; Cho, N.H.; Min, J.; Lee, M.H. Rapid, multiplexed, and nucleic acid amplification-free detection of SARS-CoV-2 RNA using an electrochemical biosensor. *Biosens. Bioelectron.* **2022**, *195*, 113649.

(7) D. Ji, D.; Guo, M.; Wu, Y.; Liu, W.; Luo, S.; Wang, X.; Kang, H.; Chen, Y.; Dai, C.; Kong, D.; Ma, H.; Liu, Y.; Wei, D. Electrochemical detection of a few copies of unamplified SARS-CoV-2 nucleic acids by a self-actuated molecular system. *J. Am. Chem. Soc.* **2022**, *144* (30), 13526-13537.

(8) Lomae, A.; Preechakasedkit, P.; Hanpanich, O.; Ozer, T.; Henry, C.S.; Maruyama, A.; Pasomsub, E.; Phuphuakrat, A.; Rengpipat, S.; Vilaivan, T.; Chailapakul, O.; Ruecha, N.; Ngamrojanavanich, N. Label free electrochemical DNA biosensor for COVID-19 diagnosis. *Talanta* **2023**, *253*, 123992.
